# Suzuki–Miyaura Catalyst-Transfer Polycondensation of Triolborate-Type Carbazole Monomers

**DOI:** 10.3390/polym13234168

**Published:** 2021-11-28

**Authors:** Saburo Kobayashi, Mayoh Ashiya, Takuya Yamamoto, Kenji Tajima, Yasunori Yamamoto, Takuya Isono, Toshifumi Satoh

**Affiliations:** 1 Graduate School of Chemical Sciences and Engineering, Hokkaido University, Sapporo 060-8628, Japan; saburou_k@eis.hokudai.ac.jp (S.K.); m_ashiya630@eis.hokudai.ac.jp (M.A.); 2 Faculty of Engineering, Hokkaido University, Sapporo 060-8628, Japan; yamamoto.t@eng.hokudai.ac.jp (T.Y.); ktajima@eng.hokudai.ac.jp (K.T.); yasuyama@eng.hokudai.ac.jp (Y.Y.)

**Keywords:** *π*-conjugated polymer, polycarbazole, catalyst-transfer polycondensation

## Abstract

Herein, we report the Suzuki–Miyaura catalyst-transfer polycondensation (SCTP) of triolborate-type carbazole monomers, i.e., potassium 3-(6-bromo-9-(2-octyldodecyl)-9*H*-carbazole-2-yl)triolborate (**M1**) and potassium 2-(7-bromo-9-(2-octyldodecyl)-9*H*-carbazole-2-yl) triolborate (**M2**), as an efficient and versatile approach for precisely synthesizing poly[9-(2-octyldodecyl)-3,6-carbazole] (3,6-PCz) and poly[9-(2-octyldodecyl)-2,7-carbazole] (2,7-PCz), respectively. The SCTP of triolborate-type carbazole monomers was performed in a mixture of THF/H_2_O using an initiating system consisted of 4-iodobenzyl alcohol, Pd_2_(dba)_3_•CHCl_3_, and *t*-Bu_3_P. In the SCTP of **M1**, cyclic by-product formation was confirmed, as reported for the corresponding pinacolboronate-type monomer. By optimizing the reaction temperature and reaction time, we successfully synthesized linear end-functionalized 3,6-PCz for the first time. The SCTP of **M2** proceeded with almost no side reaction, yielding 2,7-PCz with a functional initiator residue at the *α*-chain end. Kinetic and block copolymerization experiments demonstrated that the SCTP of **M2** proceeded in a chain-growth and controlled/living polymerization manner. This is a novel study on the synthesis of 2,7-PCz via SCTP. By taking advantage of the well-controlled nature of this polymerization system, we demonstrated the synthesis of high-molecular-weight 2,7-PCzs (*M*_n_ = 5–38 kg mol^−1^) with a relatively narrow *Ð*_M_ (1.35–1.48). Furthermore, we successfully synthesized fluorene/carbazole copolymers as well as 2,7-PCz-containing diblock copolymers, demonstrating the versatility of the present polymerization system as a novel synthetic strategy for well-defined polycarbazole-based materials.

## 1. Introduction

*π*-Conjugated polymers have attracted immense attention owing to their potential as electroactive and photoactive materials for fabricating various organic electronic devices [1,2] such as organic light-emitting diodes (OLEDs) [3,4], organic field-effect transistors (OFETs) [5,6], organic photovoltaics (OPVs) [7,8], and organic memory devices [9,10,11]. Most *π*-conjugated polymers investigated thus far consisted of thiophene [12,13,14,15,16], phenylene [17,18,19], and fluorene [20,21,22,23] units, which have been accessed through the step-growth polycondensations using cross-coupling reactions, such as Suzuki–Miyaura and Kumada–Corriu coupling. However, precise control of the molecular weight, dispersity (*Ð*_M_), and end group of the *π*-conjugated polymers has been challenging due to the step growth nature of the polymerization. On the other hand, Yokozawa and McCullough established chain-growth-type catalyst-transfer polycondensation based on Kumada coupling and Suzuki–Miyaura coupling in 2001 [24], 2005 [25], and 2006 [26], respectively. This novel polymerization mechanism opened the access to a range of *π*-conjugated polymers with a predictable molecular weight, low *Ð*_M_, and defined end groups.

Among the approaches to give *π*-conjugated polymers, catalyst transfer polymerization for polycarbazoles has not been extensively studied. Polycarbazoles can be distinguished based on the bonding positions of repeating units [27]. Poly(*N*-alkyl-3,6-carbazole)s (3,6-PCz), with an *m*-phenylene-like configuration, have been applied to OLEDs, flash memory devices, and devices utilizing thermally activated delayed fluorescence [28,29,30,31,32]. In contrast, poly(*N*-alkyl-2,7-carbazole)s (2,7-PCz), possessing a *p*-phenylene-like configuration, have been used in OLEDs and OPV devices [33,34,35,36,37,38]. Despite their promising potential in many applications, the synthesis of polycarbazoles relies on the polycondensation of symmetric bifunctional monomers (i.e., A−A + B−B type reaction, where the A and B mean complementary reactive groups) [39,40]. However, there are only a few examples of chain-growth-type catalyst transfer polycondensation for synthesizing poly(*N*-alkyl-carbazole)s [41]. Tan et al. first reported the synthesis of poly(*N*-heptadecan-2,7-carbazole) and poly(*N*-octyl-3,6-carbazole) via Kumada catalyst-transfer polycondensation (KCTP) [42]. However, the poor functional group tolerance of the Grignard-type monomers used in KCTP limited the application of functional initiators to introduce reactive functional groups on the chain end. In contrast, due to the good functional group tolerance of organoboron species, Suzuki–Miyaura catalyst-transfer polycondensation (SCTP) is highly attractive for the precise synthesis of poly(*N*-alkyl-carbazole)s with functional end groups. Jager et al. reported the synthesis of an end-functionalized poly(*N*-octyl-3,6-carbazole) via the SCTP of a pinacolboronate-type monomer [43]. However, a chain transfer reaction occurred during polymerization, leading to the formation of macrocyclic by-products and polymers with undesired end groups. To the best of our knowledge, the SCTP synthesis of 2,7-PCz has never been reported. This offers room to further investigate control of the SCTP to precisely synthesize 3,6- and 2,7-PCzs with desired molecular weights, narrow *Ð*_M_, and end groups. Accordingly, we focused on triolborate-type monomers, which possess higher nucleophilicity than conventional pinacolboronate-type monomers. Triolborate salts are known to show higher reactivity than other organoboron reagents [44,45], enabling reactions with heteroaromatic and alkyl compounds [46,47,48], which are typically inactive in Suzuki–Miyaura coupling. In addition, triolborate salts show good solubility in common organic solvents and are air-/water-stable, and hence, they can be applied for Suzuki–Miyaura coupling without the addition of water and a base. By utilizing these characteristics, we recently reported the SCTP of a triolborate salt-type fluorene monomer, which successfully compensated for the shortcomings encountered in the SCTP of conventional pinacolboronate fluorene monomers [49].

In this study, we investigated the SCTP of triolborate salt carbazole monomers, i.e., potassium 3-(6-bromo-9-(2-octyldodecyl)-9*H*-carbazole-2-yl) triolborate (**M1**) and potassium 2-(7-bromo-9-(2-octyldodecyl)-9*H*-carbazole-2-yl) triolborate (**M2**), with the aim of establishing a synthetic approach for well-defined 3,6-PCzs and 2,7-PCzs. We successfully achieved the selective synthesis of end-functionalized 3,6-PCz by the SCTP of **M1** at low temperatures to suppress the chain transfer reaction. The SCTP of **M2** proceeded in chain growth and a controlled/living polymerization mechanism to produce 2,7-PCzs with controlled molecular weights (5080–37,900 g mol^−1^) and a relatively narrow *Ð*_M_ (1.35–1.48). Using the established SCTP conditions, we successfully synthesized random copolymers of *N*-alkyl-3,6- and 2,7-carbazoles, random copolymers of *N*-alkyl-carbazole and 9,9-dialkylfluorene, and 2,7-PCz-containing block copolymers.

## 2. Materials and Methods

The Materials and Methods are described in the Supplementary Materials.

## 3. Results and Discussion

### 3.1. SCTP of M1 to Produce 3,6-PCz

**Scheme 1:**
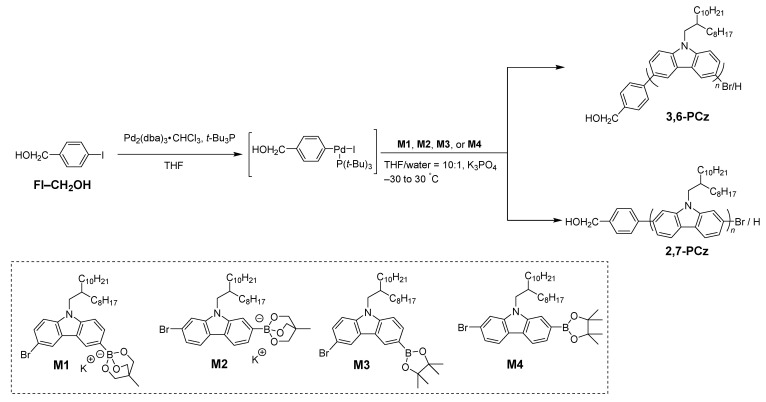
Synthesis scheme for the end-functionalized PCzs based on the SCTP of triolborate-type carbazole monomers.

We first investigated the synthesis of end-functionalized poly[9-(2-octyldodecyl)-3,6-carbazole] (3,6-PCz) with a narrow dispersity (*Đ*_M_). Recently, Jager et al. demonstrated the synthesis of 3,6-PCzs by the SCTP of a pinacol boronate-type monomer, using bromobenzene derivative as the initiator [43]. However, a significant amount of macrocyclic byproduct was generated owing to the chain transfer reaction, in addition to the desired linear polymer obtained from the bromobenzene derivative. We hypothesized that the chain transfer reaction can be suppressed by using triolborate salt monomers because of its high reactivity even at low temperatures, resulting in suppressed cyclization side reactions (Scheme 1).

**Figure 1:**
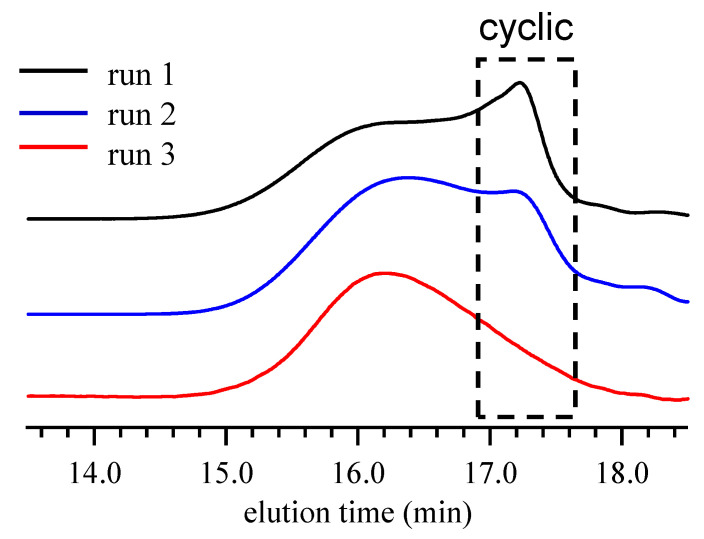
SEC traces of 3,6-PCzs obtained from runs 1, 2, and 3 in Table 1).

**Figure 2:**
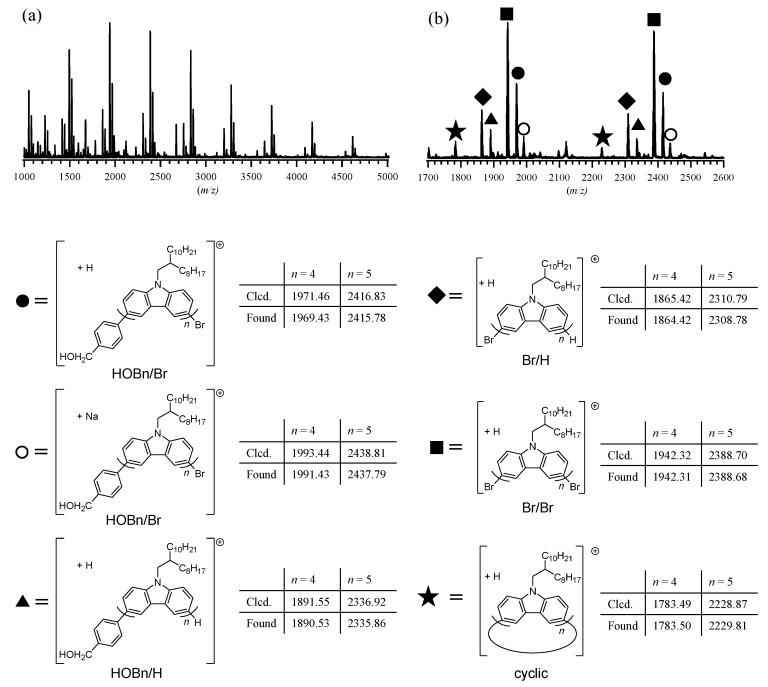
MALDI-TOF mass spectral analysis of **HOCH_2_−3,6-PCz** obtained from run 1 (Table 1). (**a**) MALDI-TOF mass spectrum. (**b**) Expanded spectrum ranging from 1700 to 2600 Da.

We first attempted the polymerization of potassium 3-(6-bromo-9-(2-octyldodecyl)-9*H*-carbazole-2-yl)triolborate (**M1**) using an initiating system that consisted of 4-iodobenzyl alcohol (**FI−CH_2_OH**), Pd_2_(dba)_3_•CHCl_3_, and *t*-Bu_3_P, with an [**M1**]_0_/[**FI−CH_2_OH**]_0_/[Pd_2_(dba)_3_•CHCl_3_]/[*t*-Bu_3_P]/[K_3_PO_4_] ratio of 15/1/0.3/2.2/1.5 at 30 °C in a mixture of THF/water (10:1; *v*/*v*, [**M1**]_0_ = 10 mmol L^−1^), based on the previously established conditions for polyfluorene synthesis [49]. Although SCTP proceeded to afford 3,6-PCz (*M*_n,SEC_ = 5500 g mol^−1^), the size-exclusion chromatography (SEC) analysis revealed a bimodal distribution with a dispersity (*Đ*_M_) of 1.32 (run 1, Table 1, Figure 1). The structure of the 3,6-PCz was investigated using matrix-assisted laser desorption/ionization time-of-flight mass spectroscopy (MALDI-TOF MS). As shown in Figure 2, the MALDI-TOF mass spectrum exhibited six series of peaks with a regular interval of 445.47 Da, corresponding to 9-(2-octyldodecyl) carbazole repeating units. One of the observed series of peaks was assigned to HOCH_2_−3,6-PCz with a phenylmethanol residue at the *α*-chain end and a bromine atom at the *ω*-chain end (BnOH/Br); for example, the peak of 1969.43 at *m*/*z* matched well with the theoretical mass for the 4-mer of HOCH_2_−3,6-PCz ([M + H]^+^ = 1971.46 Da). However, peaks corresponding to 3,6-PCz with hydrogen and bromine ends (Br/H; e.g., *m*/*z* of 1864.42 Da), bromine at both ends (Br/Br; e.g., *m*/*z* of 1942.31 Da), and cyclic 3,6-PCz (e.g., *m*/*z* = 1783.50 Da) were observed, revealing the uncontrolled polymerization nature.

According to a report by Jager et al. [43], the sharp SEC elution peaks observed in the lower-molecular-weight region and the relatively broad elution peak at high molecular weight in Figure S1 most likely arise from the cyclic by-product and linear polymer, respectively. The MALDI-TOF mass spectrum of the high-molecular-weight side, which was isolated by preparative SEC, predominantly showed peaks corresponding to 3,6-PCz, with a phenylmethanol residue at the end (Figure S1). Nevertheless, these results suggest that the chain transfer reaction occurred significantly, even with the triolborate-type monomer under this polymerization condition.

To inhibit the chain transfer reaction, we optimized the polymerization conditions for **M1**. Hong et al. reported the synthesis of polyfluorene with a narrow *Đ*_M_ by SCTP with the addition of extra *t*-Bu_3_P [50]. Therefore, we conducted the polymerization of **M1** by adding 4.4 equiv. of *t*-Bu_3_P with respect to **FI−CH_2_OH**, without changing any other factors (run 2, Table 1). As a result, the SEC elution peak corresponding to the cyclic by-product was reduced compared to that in run 1, implying that the chain transfer reaction was suppressed to some extent (Figure 1). We also examined the polymerization by changing the phosphine ligand (e.g., dppp, XPhos, and RuPhos), but no positive results were obtained. Next, polymerization was carried out by lowering the reaction temperature (−30 °C and −10 °C) to further reduce the unwanted chain transfer reaction. For polymerization at −30 °C (run 4), **M1** precipitated during SCTP. In contrast, the polymer given at −10 °C (run 3) exhibited a nearly monomodal SEC elution peak with a narrow dispersity of 1.19 (Figure 1). Consequently, lowering the reaction temperature was found to be an effective method to suppress the chain transfer reaction as well as cyclic by-product formation. For comparison, we examined the polymerization of a conventional pinacolboronate-type carbazole monomer, 3-(6-bromo-9-(2-octyldodecyl)-9*H*-carbazole-2-yl)4,4,5,5-tetramethyl-1,2,3dioxaborolane (**M3**), under the same conditions as for **M1**. However, no polymerization was observed at −10 °C (run 5, Table 1, Figure S3). This result demonstrates that the SCTP of the triolborate salt monomer at low temperatures is essential for the controlled synthesis of 3,6-PCz.

**Figure 3:**
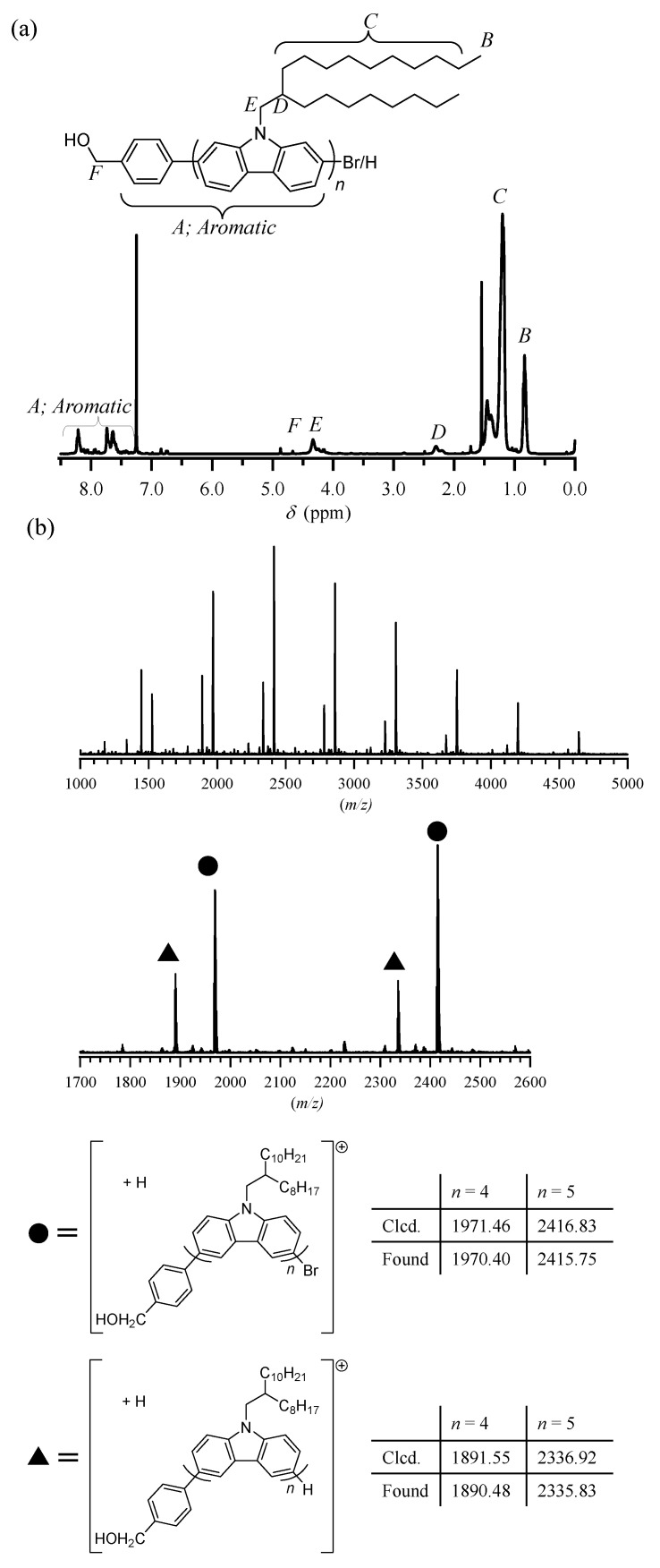
^1^H NMR and MALDI-TOF mass spectral analysis of **HOCH_2_−3,6-PCz** obtained from run 3 (Table 1). (**a**) ^1^H NMR in CDCl_3_ (400 MHz). (**b**) MALDI-TOF mass spectrum with peak assignments.

The structure of 3,6-PCz obtained at −10 °C (run 3) was characterized in detail by NMR and mass spectral analyses. In the ^1^H NMR spectrum (Figure 3a), the major signals were successfully assigned to the 3,6-PCz backbone (8.65–7.31 ppm: proton A) and 2-octyldodecyl side chain (2.11 ppm: proton B, 4.12 ppm: proton C, 1.82–0.75 ppm: proton D and E), which is consistent with the reported ^1^H NMR data for poly[9-(2-octyl)-3,6-carbazole] synthesized via SCTP [43]. More importantly, a minor signal due to the benzyl proton (proton F) in the *α*-chain end group was observed at 4.75 ppm, indicating the initiation from **FI−CH_2_OH**. Thus, the obtained product was identified as the expected poly[9-(2-octyldodecyl)-3,6-carbazole] possessing a phenylmethanol residue at the *α*-chain end (**HOCH_2_−3,6-PCz**). The number-average molecular weight (*M*_n,NMR_) of the **HOCH_2_−3,6-PCz** (run 3), determined based on end group analysis of the ^1^H NMR spectrum, was 6700 g mol^−1^. In order to confirm the end-group fidelity, we performed MALDI-TOF MS analysis on this sample. The MALDI-TOF mass spectrum showed two series of peaks with a regular interval of 445.47 Da, corresponding to 9-(2-octyldodecyl) carbazole repeating units (Figure 3b). The peaks indicated by filled circles (●) were assignable to **HOCH_2_−3,6-PCz** possessing a phenylmethanol residue at the *α*-chain end and a bromine atom at the *ω*-chain end (BnOH/Br); for example, the peak at *m*/*z* of 1970.40 showed good agreement with the theoretical mass for the 4-mer ([M + H]^+^ = 1970.46 Da). The peaks indicated by filled triangles (▲) were assignable to **HOCH_2_−3,6-PCz** possessing a phenylmethanol residue at the *α*-chain end and a hydrogen atom *ω*-chain end (BnOH/H). Overall, the SCTP of **M1** at low temperature enables better control, yielding narrowly dispersed 3,6-PCzs with sufficient end-group fidelity.

**Figure 4:**
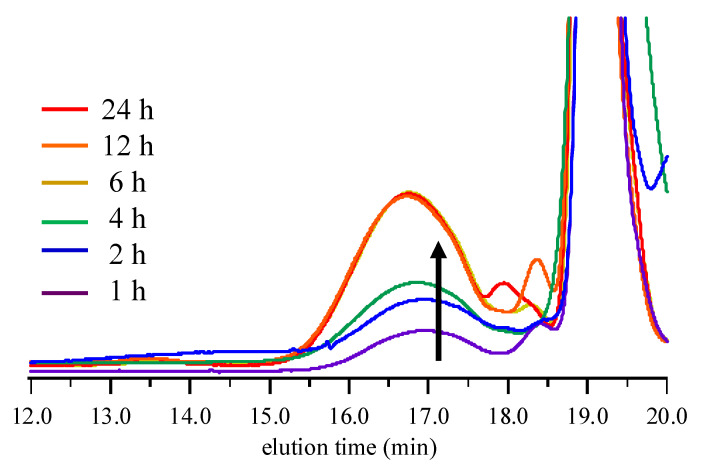
SEC traces of crude aliquot collected from the polymerization mixture of **M1** at selected times.

**Figure 5:**
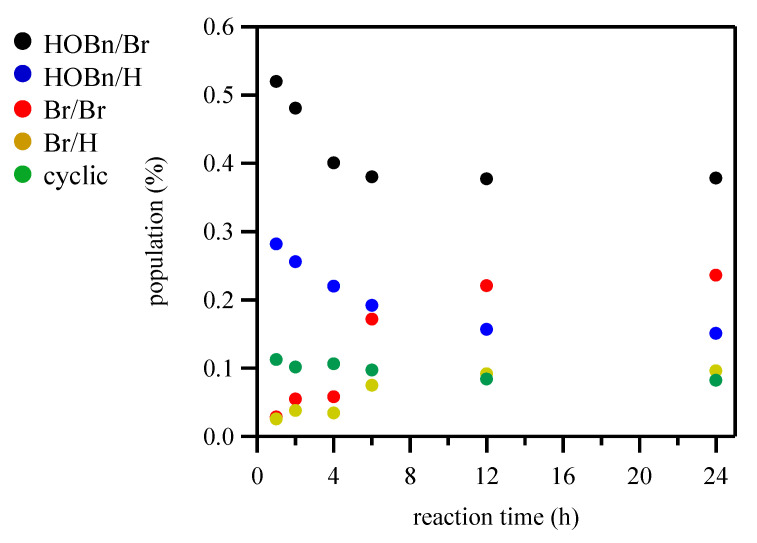
Evolution of 3,6-PCz species with different chain ends over time, as determined by MALDI-TOF MS analysis.

**Scheme 2:**
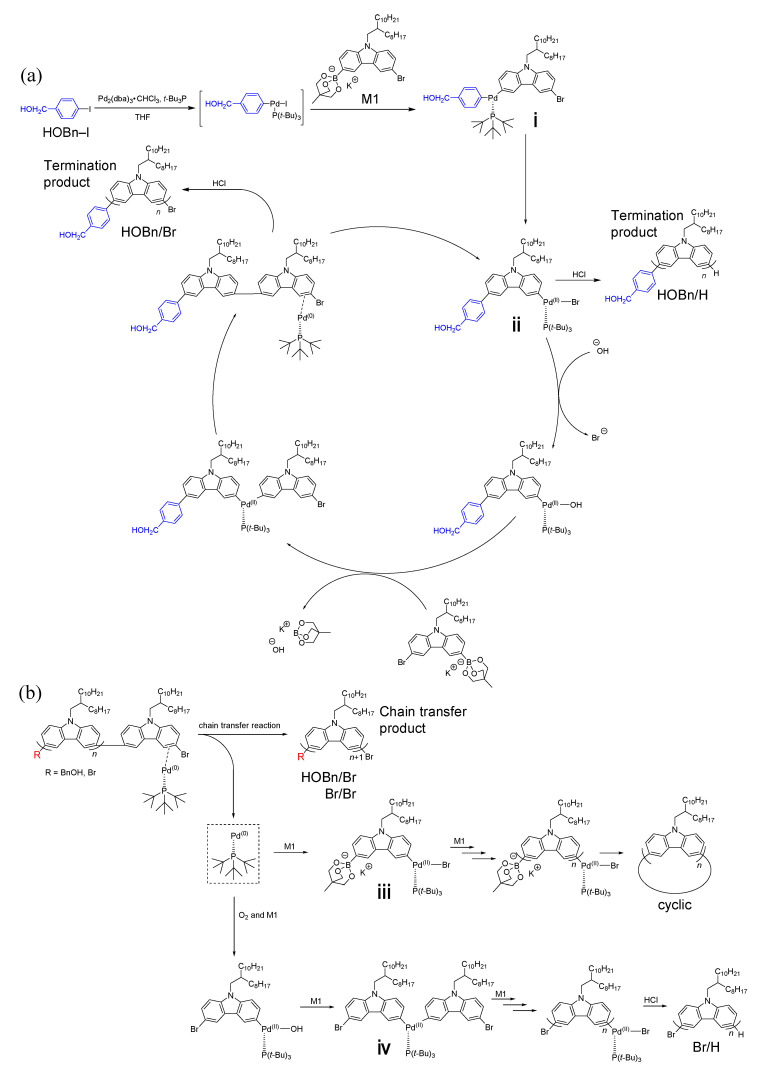
(**a**) Polymerization mechanism of 3,6-PCz. (**b**) Plausible mechanisms for the formation of 3,6-PCzs with different chain end structures and cyclic 3,6-PCz.

To understand the polymerization properties of **M1**, we evaluated the time-dependent change of the reaction product via the SEC and MALDI-TOF MS analysis of the crude aliquot quenched by the addition of HCl (1 mol L^−1^). SEC did not confirm an increase in molecular weight over time, as expected for chain growth polycondensation (Figure 4). This suggests that the polymerization of **M1** does not proceed in a perfect living fashion, even with the best polymerization conditions. It is reasonable to deduce that various side reactions occur in this polymerization system. MALDI-TOF MS analysis revealed a complex polymerization behavior. From the beginning of polymerization to 5 h, 3,6-PCz with HOBn/Br (*m*/*z* of 1969.3 Da) and HOBn/H (*m*/*z* of 1969.3 Da) chain end structures were the major products, suggesting that polymerization was initiated from the palladium 4-iodobenzyl alcohol[tris(1,1-dimethylethyl)phosphine] (iodobenzyl alcohol-Pd) complex, as expected. However, when polymerization continued thereafter, the populations of 3,6-PCz with Br/Br (*m*/*z* of 1943.1 Da) and Br/H (*m*/*z* of 1863.3 Da) chain end structures increased over time (Figure 5 and Figure S2). This complex polymerization behavior can be explained as shown in Scheme 2. The first step of polymerization involves the transmetallation between the initiator and monomer to yield the Pd complex (**ⅰ**) when the Pd(0) species, which are generated by reductive elimination, are intramolecularly shifted to the carbon–bromine bond at the end of the same molecule. Polymerization proceeds via chain growth from (**ⅱ**), resulting in the formation of 3,6-PCz with a phenylmethanol residue at the initiation end (HOBn/Br or HOBn/H) (Scheme 2a). However, when the Pd(0) species does not shift to the bromine end in the same molecule but intramolecularly shifts to the new monomer, a new initiating species (**ⅲ**) is generated [51,52,53,54]. The initiating species (**ⅲ**) generates 3,6-PCz with a triolborate residue at the *α*-chain end and bromine atom at the *ω*-chain end, which results in the formation of the cyclic by-product though the intramolecular reaction. Meanwhile, when the Pd catalyst is oxidized by a trace amount of oxygen [55,56], the homocoupling of two monomer molecules (**ⅳ**) produces another initiating species, which results in the formation of 3,6-PCz at the brominated *α*-chain end (Br/Br and Br/H) (Scheme 2b).

Overall, even the polymerization of the triolborate-type monomer **M1** did not proceed in a perfect fashion. Nevertheless, we successfully obtained 3,6-PCz with the desired initiator residue virtually without contamination of the cyclic by-product, by optimizing the polymerization temperature and time.

### 3.2. SCTP of M2 to Produce 2,7-PCz

Next, we investigated the polymerization of potassium 2-(7-bromo-9-(2-octyldodecyl)-9*H*-carbazole-2-yl)triolborate (**M2**). The initial attempt was carried out using an initiating system that consisted of **FI−CH_2_OH**, Pd_2_(dba)_3_•CHCl_3_, and *t*-Bu_3_P with an [**M2**]_0_/[**FI−CH_2_OH**]_0_/[Pd_2_(dba)_3_•CHCl_3_]/[*t*-Bu_3_P]/[K_3_PO_4_] ratio of 15/1/0.3/2.2/1.5 at 30 °C in a mixture of THF/water (10:1; *v*/*v*, [**M2**]_0_ = 10 mmol L^−1^) (Scheme 1; run 6 in Table 1), according to the best polymerization conditions for potassium 2-(7-bromo-9,9-dihexyl-9*H*-fluorene-2-yl)triolborate [49]. The SCTP of **M2** successfully proceeded to afford a polymer with *M*_n,SEC_ = 3700 g mol^−1^ and *Đ*_M_ = 1.23. To study the effect of the polymerization temperature, we carried out SCTP at −30 °C and −10 °C while fixing the other reaction parameters. At −30 °C (run 7), the monomer and product precipitated during the SCTP. Meanwhile, at −10 °C (run 8), a low monomer conversion was observed even after polymerization for 24 h. Consequently, it became apparent that **M2** should be performed at 30 °C. For comparison, we examined the SCTP of a conventional pinacolboronate-type monomer, i.e., 2-(7-bromo-9-(2-octyldodecyl)-9*H*-carbazole-2-yl)4,4,5,5-tetramethyl-1,2,3dioxaborolane (**M4**) under the comparable condition (run 9). As a result, no polymerization was observed even after 24 h. This result demonstrates the advantage of using a triolborate salt monomer for the synthesis of 2,7-PCz.

**Figure 6:**
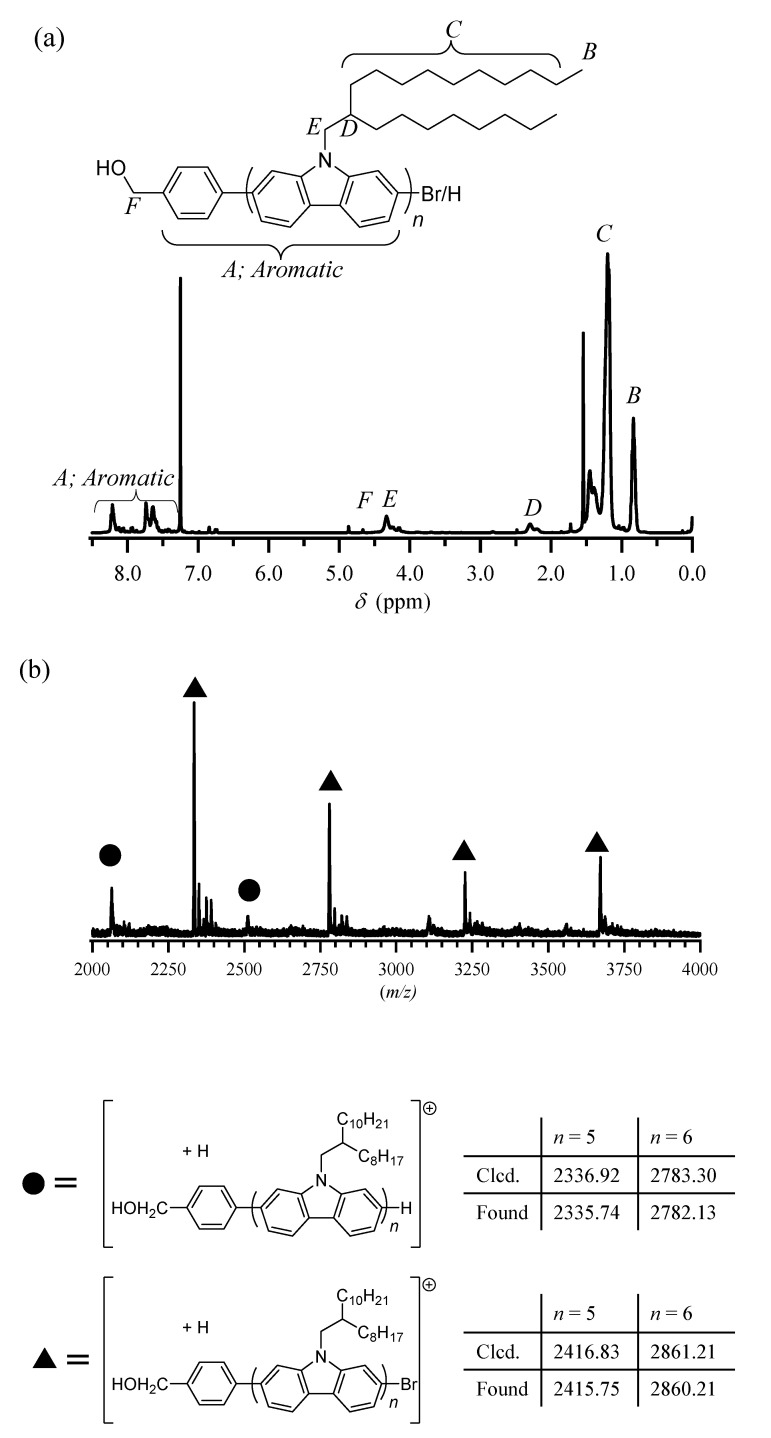
^1^H NMR and MALDI-TOF mass spectral analysis on **HOCH_2_−2,7-PCz** obtained from run 6 (Table 1) (**a**) ^1^H NMR in CDCl_3_ (400 MHz). (**b**) MALDI-TOF mass spectrum with peak assignments.

Then, the structure of the obtained 2,7-PCz was characterized in detail by NMR and MALDI-TOF mass spectral analyses. Figure 6a shows the ^1^H NMR spectrum of the product obtained from run 6. The major observed signals were reasonably assignable to the 2,7-PCz backbone (8.68–7.33 ppm: proton A) and 2-octyldodecyl side chain (2.12 ppm: proton B, 4.14 ppm: proton C, 1.82–0.73 ppm: proton D and E), which is consistent with the reported ^1^H NMR data for poly[*N*-heptadecan-2,7-carbazole] synthesized via KCTP [42]. More importantly, a minor signal at 4.75 ppm was assigned to the benzyl proton at the *α*-chain end (proton E), which confirmed the successful installation of the functional end group. Therefore, the obtained product was ascribed to the expected poly[9-(2-octyldodecyl)-2,7-carbazole] possessing a phenylmethanol residue at the *α*-chain end (**HOCH_2_−2,7-PCz**). The *M*_n,NMR_ of **HOCH_2_−2,7-PCz** (run 6) was calculated to be 5080 g mol^−1^ by end-group analysis of the ^1^H NMR spectrum. In order to further confirm the end-group fidelity, we performed MALDI-TOF MS analysis on the product from run 6. The MALDI-TOF mass spectrum mainly exhibited two series of peaks showing a regular interval of 445.4 Da corresponding to 9-(2-octyldodecyl) carbazole repeating units (Figure 6b). The peaks denoted by filled triangles (▲) were assignable to **HOCH_2_−2,7-PCz** possessing a phenylmethanol residue at the *α*-chain end and a bromine atom at the *ω*-chain end. For example, the peak at *m*/*z* = 2415.75 matched with the theoretical mass for the 5-mer (BnOH/Br; [M + H]^+^ = 2416.83 Da). The peaks denoted by filled circles (●) were assigned to **HOCH_2_−2,7-PCz** possessing a phenylmethanol residue at the *α*-chain end and a hydrogen atom at the *ω*-chain end (BnOH/H; [M + H]^+^ = 2336.92 Da). Overall, in contrast to the case of **M1**, the SCTP of **M2** proceeded in a controlled/living fashion to give narrowly dispersed 2,7-PCzs with good end-group fidelity.

**Figure 7:**
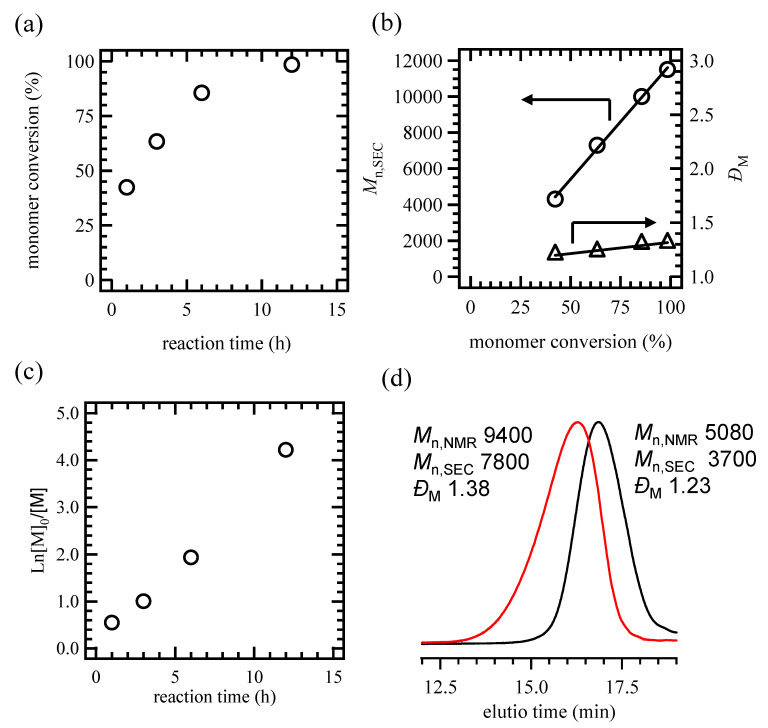
(**a**) Time-conversion plot in the SCTP of **M2** ([**M2**]_0_/[**FI−CH_2_OH**]_0_/[Pd_2_(dba)_3_•CHCl_3_]/[*t*-Bu_3_P]/[K_3_PO_4_] = 15:1:0.3:2.2:1.5; solvent, THF/water (10:1, *v*/*v*); temp., 30 °C). (**b**) Dependence of *M*_n,SEC_ and *Ð_M_* on the monomer conversion. (**c**) Kinetic plots for SCTP. (**d**) SEC traces of 2,7-PCz (black line) and 2,7-PCz-*b*-PF obtained after the second polymerization (red line).

**Scheme 3:**
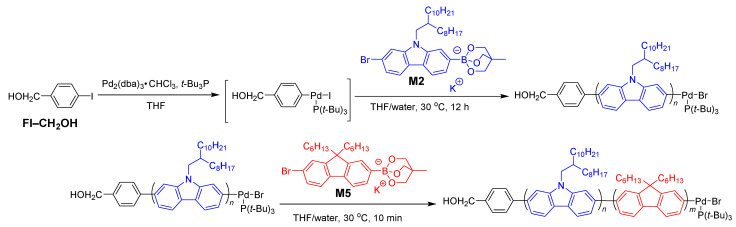
Block copolymerization experiment to confirm controlled/living nature in the SCTP of **M2**.

In order to demonstrate the chain growth and controlled/living polymerization behaviors in the SCTP of **M2**, we carried out kinetic and block copolymerization experiments (Scheme 3). For the kinetic study, the polymerization of **M2** using **FI−CH_2_OH** was performed in a mixture of THF/water (10:1, *v*/*v*, [**M2**]_0_ = 10 mmol L^−1^) at 30 °C with an [**M2**]_0_/[**FI−CH_2_OH**]_0_/[Pd_2_(dba)_3_•CHCl_3_]/[*t*-Bu_3_P]/[K_3_PO_4_] ratio of 30/1/0.3/2.2/1.5. As shown in Figure 7a, polymerization proceeds smoothly, and >98% of the monomer is consumed within 12 h. Figure 7b reveals that the *M*_n,SEC_ of the obtained 2,7-PCz linearly increases with the increase in the monomer conversion, while the *Ð*_M_ values remain narrow (*Ð*_M_ < 1.4). This is strong evidence for the chain-growth mechanism of the SCTP. The kinetic plot indicates a distinct first-order kinetic behavior for this polymerization system. According to slope of the kinetic plot, the rate constant was estimated to be 5.8 s^−1^ mmol L^−1^ (Figure 7c). A block copolymerization experiment was further carried out to prove the controlled/living nature of the propagating end of 2,7-PCz. **M2** was first polymerized for 12 h with an [**M2**]_0_/[**FI−CH_2_OH**]_0_/[Pd_2_(dba)_3_•CHCl_3_]/[*t*-Bu_3_P]/[K_3_PO_4_] ratio of 15/1/0.3/2.2/1.5, to yield an intermediate 2,7-PCz with an *M*_n,SEC_ of 3700 g mol^−1^ and *Ð_M_* of 1.23. After confirming full monomer conversion, 15 equiv. of potassium 2-(7-bromo-9,9-dihexyl-9*H*-fluorene-2-yl)triolborate (**M5**) was added for chain extension, which afforded 2,7-PCz-*b*-PF with *M*_n,NMR_ of 9400 g mol^−1^ and a *Ð_M_* of 1.38. The SEC traces of the products before and after block copolymerization are shown in Figure 7d, in which the SEC elution peak showed clear shift toward the high-molecular-weight side upon the addition of the second monomer. This result verified a truly controlled/living propagating end, which led to chain extension by the second monomer addition. Overall, the aforementioned results successfully support the chain-growth and controlled/living polymerization nature of the SCTP of **M2**.

**Figure 8:**
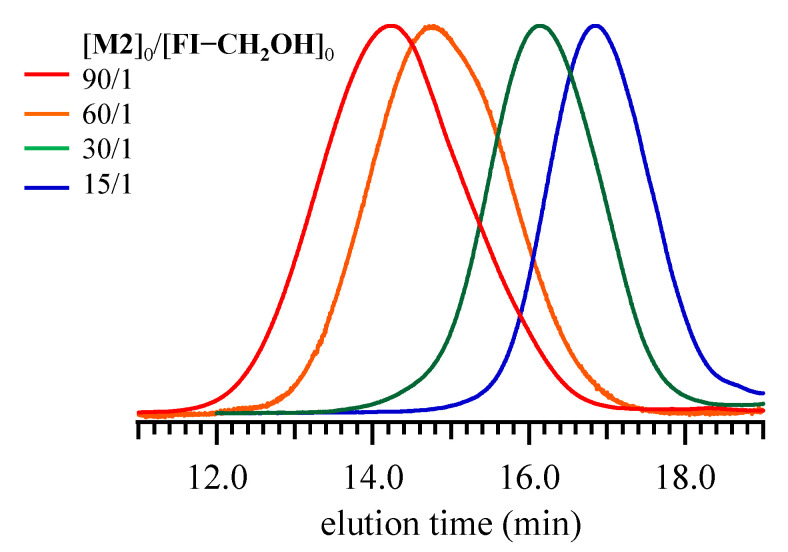
SEC traces of **HOCH_2_−2,7-PCz** obtained with different [**M2**]_0_/[**FI−CH_2_OH**]_0_ ratios.

With the optimized polymerization conditions, the SCTP of **M2** was performed with different [**M2**]_0_/[**FI−CH_2_OH**]_0_ ratio (30/1 for run 10, 60/1 for run 11, and 90/1 for run 12) to synthesize 2,7-PCzs with different molecular weights. Thus, 2,7-PCzs with *M*_n,SEC_ and *Đ*_M_ values of 8600–32,000 g mol^−1^ and 1.35–1.48, respectively (runs 10–12, Table 1, *M*_n,NMR_; 5080–37,900 g mol^−1^) were successfully obtained (Figure 8). To the best of our knowledge, 2,7-PCz obtained from run 12 (*M*_n,SEC_ of 37,900 g mol^−1^) had the highest molecular weight among the poly[9-(2-octyldodecyl)-2,7-carbazoles] prepared by chain-growth polycondensation.

### 3.3. Random Copolymerization of Triolborate-Type Carbazole and Fluorene Monomers

**Figure 9:**
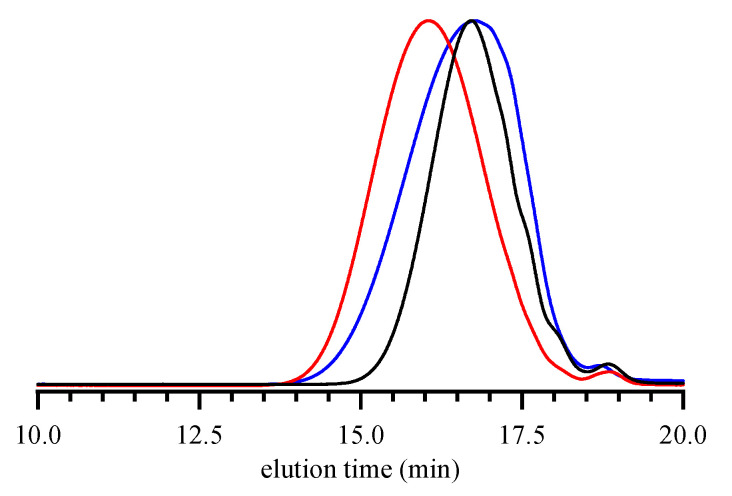
SEC traces of 2,7-PCz-*co*-3,6-PCz (black curve), PF-*co*-2,7-PCz (red curve), and PF-*co*-3,6-PCz (blue curve).

**Figure 10:**
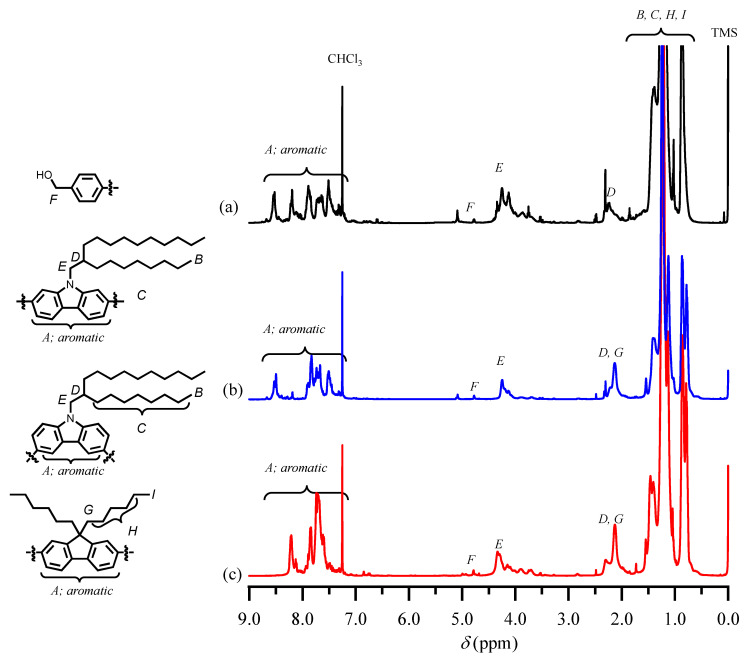
^1^H NMR spectra of (**a**) 2,7-PCz-*co*-3,6-PCz, (**b**) PF-*co*-3,6-PCz, and (**c**) PF-*co*-2,7-PCz (400 MHz, CDCl_3_).

**Scheme 4:**
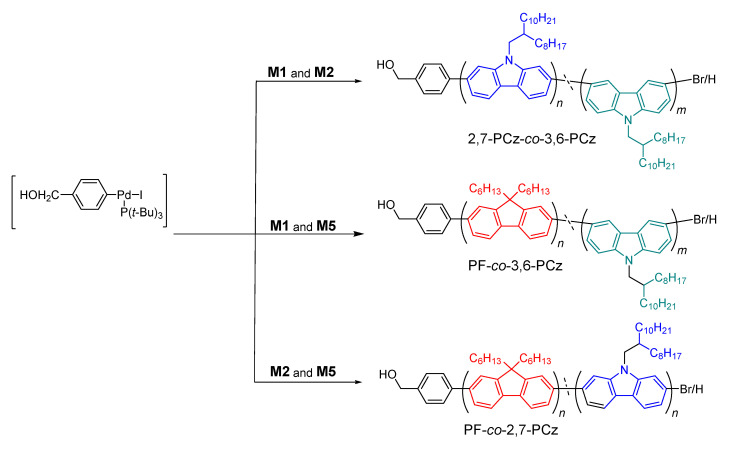
Random copolymerization of **M1**/**M2**, **M1**/**M5**, and **M2**/**M5**.

Recently, conjugated copolymers based on carbazole or fluorene groups with varying connectivity, such as at the 2,7 or 3,6 positions, were found to be useful for the extraction of single-walled carbon nanotubes [57]. Therefore, we are interested in testing our SCTP system for the synthesis of poly(carbazole-*co*-fluorene)s (Scheme 4). First, we examined the random copolymerization of **M1** and **M2** using the initiating system that consisted of **FI−CH_2_OH**, Pd_2_(dba)_3_•CHCl_3_, and *t*-Bu_3_P, at an [**M1**]/[**M2**]/[**FI−CH_2_OH**]_0_/[Pd_2_(dba)_3_•CHCl_3_]/[*t*-Bu_3_P]/[K_3_PO_4_] ratio of 15/15/1/0.3/2.2/1.5, at 30 °C in a mixture of THF/water (10:1; *v*/*v*, [monomers]_0_ = 10 mmol L^−1^) (run 13, Table 2 run 13). SCTP proceeded to afford 2,7-PCz-*co*-3,6-PCz (*M*_n,SEC_ = 7800 g mol^−1^), which exhibited a unimodal SEC elution peak (Figure 9). The chemical structure of 2,7-PCz-*co*-3,6-PCz was identified by ^1^H NMR analysis (Figure 10). The major signals were assigned to the polycarbazole backbone (8.6–7.3 ppm: proton A) and 2-octyldodecyl side chain (2.1 ppm: proton B, 4.1 ppm: proton C, 1.8–0.7 ppm: proton D and E). More importantly, a minor signal at 4.7 ppm due to the *α*-chain end benzyl proton (proton F) was clearly observed, confirming the successful installation of the functional end group. Therefore, the product identified to be the expected copolymer possessed a phenylmethanol residue at the *α*-chain end (2,7-PCz-*co*-3,6-PCz). The ratio of the 2,7-carbazole unit to the 3,6-carbazole unit in the obtained copolymer was 14:14, which was in good agreement with the monomer feed ratio. The *M*_n,NMR_ of 2,7-PCz-*co*-3,6-PCz (run 13) was calculated to be 11,500 g mol^−1^ by end-group analysis of the ^1^H NMR spectrum.

In addition, random copolymerization was carried out for the combination of carbazole and fluorene monomers, i.e., **M1**/**M5** and **M2**/**M5** (Scheme 4). Both polymerizations homogeneously proceeded and afforded the corresponding random copolymers, i.e., PF-*co*-3,6-PCz and PF-*co*-2,7-PCz, with *Đ*_M_ values of 1.38 and 1.48, respectively. The ^1^H NMR spectra of the resulted random copolymers showed characteristic signals from both the fluorene and carbazole units (Figure 10), which confirmed the successful synthesis of random copolymers.

The thermal and photophysical properties of 3,6-PCz, 2,7-PCz, and fluorene/carbazole copolymers were investigated (see the supplementary material). Our 3,6-PCz and 2,7-PCz showed thermal and photophysical properties comparable to the reported data [39,40]. The thermal decomposition temperatures of the random copolymers were comparable to those of the constituent polymers. The UV-Vis and fluorescence spectra of the random copolymers were similar to the superposition of the spectra from the constituent homopolymers.

### 3.4. Synthesis of 2,7-PCz-Containing Diblock Copolymers Using Macroinitiators

**Figure 11:**
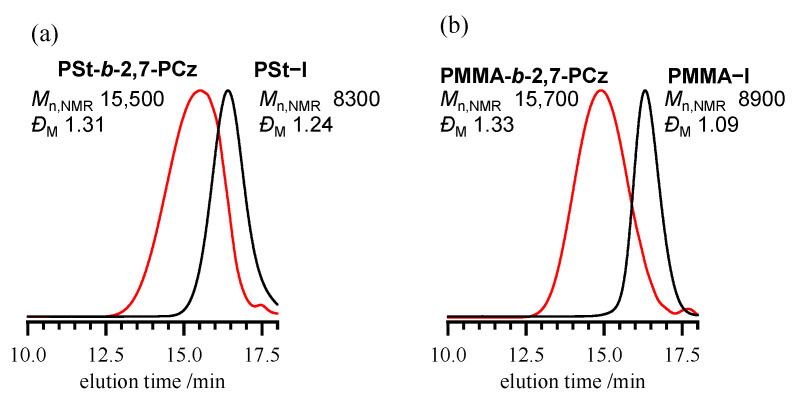
SEC traces of (**a**) PSt-*b*-2,7-PCz and (**b**) PMMA-*b*-2,7-PCz. The black and red curves represent the macroinitiators and block copolymers, respectively.

**Figure 12:**
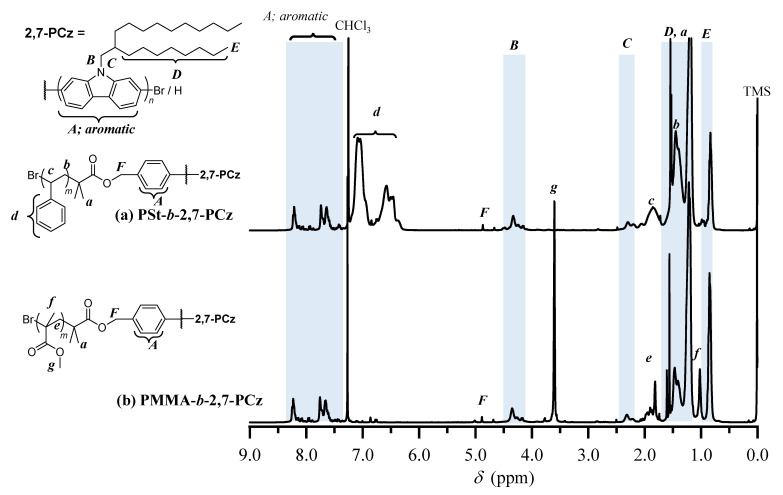
^1^H NMR spectra of (**a**) PSt-*b*-2,7-PCz and (**b**) PMMA-*b*-2,7-PCz (400 MHz, CDCl_3_).

**Scheme 5:**
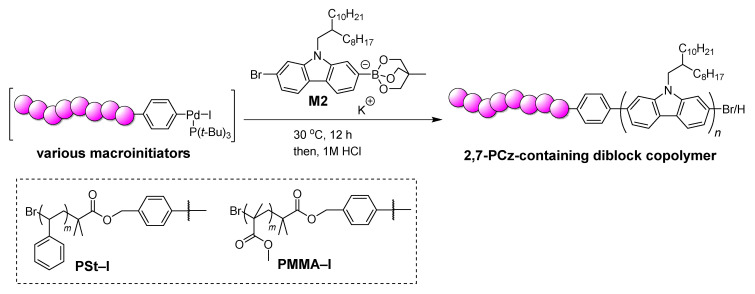
Syntheses of PSt-*b*-2,7-PCz and PMMA-*b*-2,7-PCz by SCTP using PSt−I and PMMA−I macroinitiators, respectively.

Finally, we employed iodobenzene-terminated macroinitiators for the SCTP of **M2** in order to synthesize 2,7-PCz-containing block copolymers (Scheme 5). Based on our previous study, we reduced the water content as much as possible to prevent the precipitation of the macroinitiator [49]. We first attempted SCTP with iodobenzene-terminated polystyrene, i.e., PSt−I (*M*_n,NMR_ = 8300 g mol^−1^, *Ð*_M_ = 1.24), as the macroinitiator under the optimized conditions ([PSt−I]_0_/[**M2**]_0_ = 1/15; THF/water (*v*/*v*) = 5000:1; run 16 in Table 3), giving a PSt-*b*-2,7-PCz diblock copolymer. Importantly, the SEC elution peak clearly shifted toward the shorter retention time, which revealed that **M2** polymerization was initiated from PSt−I (Figure 11). Moreover, the ^1^H NMR spectrum of the resulted product exhibited major signals attributable to both the 2,7-PCz and PSt blocks (Figure 12), confirming the successful synthesis of PSt-*b*-2,7-PCz. The *M*_n,NMR_ of the 2,7-PCz block was calculated to be 15,500 g mol^−1^. In a similar manner, SCTP with the iodobenzene-terminated poly(methyl methacrylate) macroinitiator, i.e., PMMA−I (*M*_n,NMR_ = 8900 g mol^−1^, *Ð*_M_ = 1.09), was conducted (run 17 in Table 3), which afforded PMMA-*b*-2,7-PCz with a *Đ*_M_ of 1.33 (Figure 11 and Figure 12). These results confirmed the high potential of the present SCTP system for the synthesis of end-functionalized 2,7-PCz and 2,7-PCz-containing block copolymers.

## 4. Conclusions

In this study, we investigated the SCTP of triolborate-type carbazole monomers for the first time. The SCTP of potassium 3-(6-bromo-9-(2-octyldodecyl)-9*H*-carbazole-2-yl)triolborate (**M1**) at low temperatures selectively produced end-functionalized 3,6-PCz, while suppressing the formation of cyclic by-products. The SCTP of potassium 2-(7-bromo-9-(2-octyldodecyl)-9*H*-carbazole-2-yl)triolborate (**M2**) proceeded in a chain-growth mechanism and controlled/living fashion to produce 2,7-PCzs with controlled molecular weights (5080–37,900 g mol^−1^) and a relatively narrow *Ð*_M_ (1.35–1.48). Kinetic and block copolymerization experiments revealed that the SCTP proceeded in chain growth and a controlled/living polymerization manner. Overall, we found that the precise synthesis of 3,6- and 2,7-PCzs can be realized using a triolborate-type monomer with higher nucleophilicity than those of conventionally used pinacolboronate-type monomers. In addition, the present SCTP approach enabled the synthesis of random copolymers of *N*-alkyl-3,6- and 2,7-carbazoles, random copolymers of *N*-alkyl-carbazole and 9,9-dialkylfluorene, and 2,7-PCz-containing block copolymers. We believe that this system provides easy access to a variety of functionalized polycarbazoles, eventually facilitating the development of polycarbazole-based organic devices.

## Figures and Tables

**Table 1:** SCTP of triolborate-type carbazole monomers under various conditions *^a^*.

Run	Monomer	[monomer]_0_/[FI−CH_2_OH]_0_	Temp (°C)	*M*_n,SEC_*^b^* (g mol^−1^)	*Ð_M_^b^*	Conv. (%)	Yield *^c^* (%)
1	M1	15/1	30	5500	1.32	83.2	80.6
2 *^d^*	M1	15/1	30	5900	1.30	74.2	65.2
3	M1	15/1	−10	6300	1.19	65.3	60.1
4	M1	15/1	−30	―	―	―	―
5	M3	15/1	−10	―	―	―	―
6	M2	15/1	30	3700	1.23	99.2	74.8
7	M2	15/1	−30	―	―	―	―
8	M2	15/1	−10	―	―	―	―
9	M4	15/1	30	―	―	―	―
10	M2	30/1	30	8600	1.35	98.6	74.2
11	M2	60/1	30	16,400	1.45	97.6	75.6
12	M2	90/1	30	32,000	1.48	96.4	73.2

*^a^* Polymerization condition: atmosphere, Ar; solvent, THF/water (10:1, *v*/*v*); [monomer]_0_ = 10 mmol L^−1^; [**FI−CH_2_OH**]_0_/[Pd_2_(dba)_3_•CHCl_3_]/[*t*-Bu_3_P]/[K_3_PO_4_] = 1/0.3/2.2/1.5. *^b^* Determined by SEC using PSt standards. *^c^* Isolated yields. *^d^* *t*-Bu_3_P (4.4 equiv.).

**Table 2:** Synthesis of PCz and PF-containing random copolymers *^a^*.

Run	Monomers		Random Copolymers				Yield *^d^*
	M_A_	M_B_		*M*_n,SEC_ *^b^*	*M*_n,NMR_ *^c^*	*Ð*_M_ *^b^*	(%)
				(g mol^−1^)	(g mol^−1^)		
13	**M1**	**M2**	2,7-PCz-*co*-3,6-PCz	7800	11,500	1.49	72.6
14	**M1**	**M5**	PF-*co*-3,6-PCz	9300	12,800	1.38	74.6
15	**M2**	**M5**	PF-*co*-2,7-PCz	7800	12,500	1.48	76.1

*^a^* Polymerization condition: Atmosphere, Ar; solvent, THF/water (*v*/*v*) = 10:1; [M_A_+M_B_]_0_ = 10 mmol L^−1^; [M_A_]_0_/[M_B_]_0_/[initiator]_0_/[Pd_2_(dba)_3_•CHCl_3_]/[*t*-Bu_3_P]/[K_3_PO_4_] = 15:15:1:0.3:2.2:1.5. *^b^* Determined by SEC using PSt standards. *^c^* Determined by ^1^H NMR in CDCl_3_. *^d^* Isolated yield.

**Table 3:** Polymerization of M2 using macroinitiators *^a^*.

Run	Macroinitiator			Block Copolymer			
		*M*_n,NMR_*^c^* (g mol^−1^)	*Ð*_M_ *^b^*		*M*_n,NMR_*^c^* (g mol^−1^)	*Ð*_M_ *^b^*	Yield *^d^* (%)
16	PSt−I	8300	1.24	PSt-*b*-2,7-PCz	15,500	1.31	78.6
17	PMMA−I	8900	1.09	PMMA-*b*-2,7-PCz	15,700	1.33	73.5

*^a^* Polymerization condition: atmosphere, Ar; solvent, THF/water (*v/v*) = 5000:1; [**M2**]_0_ = 10 mmol L^−1^; [**M2**]_0_/[macroinitiator]_0_/[Pd_2_(dba)_3_•CHCl_3_]/[*t*-Bu_3_P]/[K_3_PO_4_] = 15:1:1.2:8.8:1.2. *^b^* Determined by SEC using PSt standards. *^c^* Determined by ^1^H NMR in CDCl_3_. *^d^* Isolated yield.

## Supplementary Materials

The following are available online at https://www.mdpi.com/article/10.3390/polym13234168/s1. Experimental section, additional discussion for polymer syntheses, and ^1^H NMR, SEC, and MALDI-TOF MS data for various samples (PDF). Figure S1: (a) SEC trace of 3,6-PCz (run 1, Table 1) detected by RI detector (eluent, THF; flow rate, 1.0 mL min^−1^). (c) MALDI-TOF mass spectrum of 3,6-PCz obtained from run 1 (Table 1). (b and d) SEC trace and MALDI-TOF mass spectrum of 3,6-PCz obtained from run 1 after preparative SEC. Figure S2: Expanded MALDI-TOF mass spectra ranging from 1200 to 4000 Da of 3,6-PCz. Figure S3: SEC traces of crude reaction mixture for the SCTP of M3 (upper) and M4 (lower) (eluent, THF; flow rate, 1.0 mL min^−1^). Table S1: Monomer conversion, molecular weight, and dispersity at each reaction time.
