# Supplementary material for: Suzuki–Miyaura Catalyst-Transfer Polycondensation of Triolborate-Type Carbazole Monomers
Source: Polymers (Basel). 2021 Nov 28;13(23):4168. doi: 10.3390/polym13234168 (PMC8659485; doi:10.3390/polym13234168)
Supplement: Supplementary file 1 [file polymers-13-04168-s001.zip › polymers-1471072-supplementary.pdf]

Supplementary Materials

Suzuki–Miyaura Catalyst-Transfer Polycondensation of Triolborate-Type Carbazole Monomers

Authors

Saburo Kobayashi,[a] Mayoh Ashiya, [a] Takuya Yamamoto,[b] Kenji Tajima,[b] Yasunori Yamamoto,[b] Takuya Isono,[b, *] Toshifumi Satoh [b, *]

Author Affiliation

[a] Graduate School of Chemical Sciences and Engineering, Hokkaido University, Sapporo 060-8628, Japan

[b] Faculty of Engineering, Hokkaido University, Sapporo 060-8628, Japan

* To whom correspondence should be addressed: isono.t@eng.hokudai.ac.jp (T.I.) & satoh@eng.hokudai.ac.jp (T.S.)

Materials

2,7-Dibromo-9-(2-octyldecyl)-9*H*-carbazole,[1] 3,6-dibromo-9-(2-octyldecyl)-9*H*-carbazole,[2] iodobenzene-terminated polystyrene (**PSt–I**; $M_{n,NMR}$; 8,300 g mol^{-1}, $Đ_M$; 1.24),[3] 4-iodobenzyl 2-bromo-2-methylpropanoate,[3] potassium 2-(7-bromo-9,9-dihexyl-9*H*-fluorene-2-yl)triolborate,[3] and tris(dibenzylideneacetone)dipalladium(0)-chloroform adduct ($Pd_2(dba)_3$•$CHCl_3$)[4] were prepared according to reported methods.

1,3-Bis(diphenylphosphino)propane (dppp), 2-Dicyclohexylphosphino-2',4',6'-triisopropylbiphenyl (XPhos), 4-iodobenzyl alcohol, methyl methacrylate (MMA), *N*,*N*,*N*',*N*'',*N*''-pentamethyldiethylenetriamine (PMDETA), pinacol, *trans*-2-[3-(4-*tert*-butylphenyl)-2-methyl-2-propylidene]malononitrile, triisopropyl borate, and trimethylolethane were purchased from Tokyo Chemical Industry Co., Ltd. (TCI), and used as received. *n*-Butyllithium (*n*-BuLi; in *n*-hexane as 1.6 mol L^{-1} solution), potassium hydroxide (KOH), and triethylamine (Et_3N) were purchased from Kanto Chemical Co., Inc., and used as received. 2,2'-Bipyridyl, tri(*t*-butyl)phosphine (*t*-Bu_3P), and tripotassium phosphate (K_3PO_4) were purchased from Fujifilm Wako Pure Chemical Co. and used as received. Copper (I) bromide (CuBr) and 2-Dicyclohexylphosphino-2',6'-diisopropoxybiphenyl (RuPhos) were purchased from Sigma-Aldrich Co. and used as received.

Commercially-available dry-THF and dry-toluene (Kanto Chemical Co., Inc., >99.5%, water content, <0.001%) were further purified by an MBRAUN MB SPS Compact solvent purification system equipped with a MB-KOL-C column and a MB-KOL-A column, which were then directly used for the polymerizations.

Instruments

Polymerization was carried out in an MBRAUN stainless steel glovebox equipped with a gas purification system (molecular sieves and a copper catalyst) under a dry argon atmosphere (H_2O, O_2 <0.1 ppm). The moisture and oxygen contents in the glovebox were monitored by an MB-MO-SE 1 moisture sensor and an MB-OX-SE 1 oxygen sensor, respectively.

^{1}H (400 MHz) and ^{13}C NMR (100 MHz) spectra were obtained using a JEOL JNM-ECS400 instrument at 25 °C.

Size exclusion chromatography (SEC) was performed at 40 °C using a Jasco high-performance liquid chromatography system (PU-2080Plus Intelligent HPLC pump, CO-2065Plus Column oven, RI-2031Plus Intelligent RI detector, and Shodex DG-2080-54) equipped with a Shodex KF-G guard column (4.6 mm × 10 mm; particle size, 8 μm) and two Shodex KF-804 columns (linear; particle size, 7 μm; 8.0 mm × 300 mm; exclusion limit, 4×10^5) in THF at a flow rate of 1.0 mL min^{-1}. The number-average molecular weight ($M_{n,SEC}$) and dispersity ($Đ_M$) of the polymer were calculated on the basis of a polystyrene calibration.

Matrix-assisted laser desorption ionization time-of-flight mass spectrometry (MALDI-TOF MS) measurement of the polymer was carried out in the reflector mode using an ABSCIEX TOF/TOF/5800 equipped with a 337 nm nitrogen laser (3 ns pulse width). The MALDI-TOF MS samples were prepared by depositing a mixture of the polymer and matrix in THF onto a sample plate. A 1:80 (v/v) ratio of [PCz (1.0 g L^{-1} in THF)]/[*trans*-2-[3-(4-*tert*-butylphenyl)-2-methyl-2-propylidene]malononitrile (10 g L^{-1} in THF)] was used.

Thermogravimetric analysis (TGA) was performed using Hitachi STA200RV under nitrogen atmosphere. All polymer samples were heated up to 550 °C at the heating rate of 10 °C min^{-1}.

Differential scanning calorimetry (DSC) was carried out on a DSC7000X (Hitachi High-Tech Corporation) calibrated with the indium and tin standards. All the polymer samples were heated to 250 °C, cooled to 0 °C, and heated to 300 °C at the heating and cooling rates of 10 °C min^{-1} and 5 °C min^{-1}, respectively.

Absorption spectra were obtained using a JASCO V-670 spectrophotometer. Fluorescence spectra were recorded on a JASCO FP-6500H fluorescence spectrometer at 298 K. The excitation wavelength was 300 nm for all the polymer samples, which were determined by their excitation spectra. The concentration conditions for measuring the absorption and fluorescence spectroscopies in solution were 20 μg mL^{-1} and 2 μg mL^{-1} in $CHCl_3$ for all the polymer samples, respectively.

Synthesis of potassium (bromo-9-(2-octyldecyl)-9*H*-carbazole-2-yl)triolborate

Scheme S1. Synthesis of triolborate salt carbazole monomers

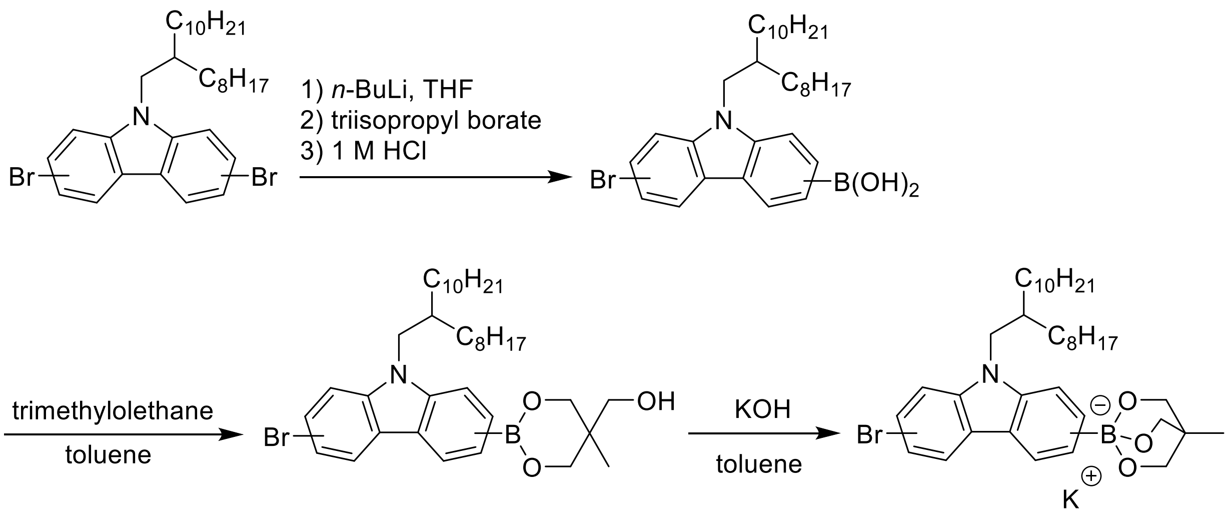

A general protocol is as follows: To a 500 mL three-necked round bottom flask, dibromo-9-alkyl-9*H*-carbazole (2,7-dibromo-9-(2-octyldecyl)-9*H*-carbazole or 3,6-dibromo-9-(2-octyldecyl)-9*H*-carbazole; 7.5 g, 12.4 mmol) was added and dried under vacuum at room temperature for 1.5 h. Dry-THF (100 mL) was introduced to this flask and cooled to −78 °C. *n*-BuLi (8.8 mL, 13.6 mmol, 1.6 mol L^{-1} in *n*-hexane) was injected slowly via a syringe, and the whole mixture was stirred for 3 h under argon atmosphere. Triisopropyl borate (4.3 mL, 18.6 mmol) was then added to this reaction mixture. The mixture was gradually brought back to room temperature and stirred for 12 h under argon atmosphere. The reaction was quenched by the addition of 1 mol L^{-1} HCl (30 mL). The solvent was removed by evaporation, and the residue was dissolved in CH_2Cl_2 and washed with brine. The organic layer was dried over Na_2SO_4 and evaporated completely. The residue was purified by silica gel column chromatography (*n*-hexane/Et_3N = 50/1 (v/v) → *n*-hexane/acetone/Et_3N = 10/3/1 (v/v/v)) to give bromo-9-alkyl-9*H*-carbazole-2-yl boronic acid as a yellow powder.

Bromo-9-alkyl-9*H*-carbazole-2-yl boronic acid (3.5 g, 6.14 mmol) and trimethylolethane (0.74 g, 6.13 mmol) were suspended in toluene (100 ml). Water was removed by azeotropic distillation for 1 h by a Dean-Stark apparatus. Then, KOH (0.41 g, 7.36 mmol) was added, and the mixture was refluxed for 2 h. The precipitate was collected by filtration, washed with water, and dried under reduced pressure to give potassium bromo-9-alkyl-9*H*-carbazole-2-yl triolborate.

Potassium 2-(7-bromo-9-(2-octyldecyl)-9*H*-carbazole-2-yl)triolborate

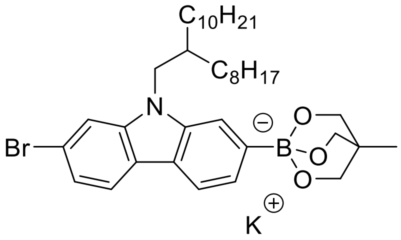

3.89 g, 89.0% yield: Yellow solid

^{1}H NMR (400 MHz, MeOD-d_4): δ (ppm) 8.08 (d, J = 7.8 Hz, 2H, Ar–H), 7.88 (s, 1H, Ar–H), 7.67 (s, 1H, Ar–H), 7.59 (d, J = 7.8 Hz, 1H, Ar–H), 7.46 (d, J = 7.3 Hz, 1H, Ar–H), 4.86 (s, 6H, −BOCH_2−), 4.14 (d, 2H, J = 7.3 Hz, −NCH_2CH−), 2.16 (m, 1H, −NCH$_2$CH−), 1.30-1.14 (m, 32H, −CH$_2$(CH_2)$_7$CH$_3$, −CH$_2$(CH_2)$_9$CH$_3$), 0.93 (s, 3H, −B(OCH$_2$)$_3$CCH_3), 0.86 (m, 6H, −(CH$_2$)$_7$CH_3, −(CH$_2$)$_9$CH_3).
^{13}C NMR (100 MHz, MeOD-d_4): δ (ppm) 141.1 (Ar), 140.8 (Ar), 130.4 (Ar), 129.9 (Ar), 129.8 (Ar), 124.3 (Ar), 123.7 (Ar), 123.4 (Ar), 119.8 (Ar), 119.6 (Ar), 114.7 (Ar), 114.3 (Ar), 72.7 (−B(OCH$_2$)$_3$−), 46.9 (−NCH$_2$CH−), 37.3 (−NCH$_2$$C$H−), 36.4 (−B(OCH$_2$)$_3$$CCH_3$), 31.8 (−CH$_2$$CH_2$(CH$_2$)$_5CH_3$, −CH$_2$$CH_2$(CH$_2$)$_7CH_3$), 29.4 (−CH$_2CH_2$($CH_2$)$_4CH_2CH_3$, −CH$_2$$CH_2$($CH_2$)$_6CH_2CH_3$), 26.2 (−CH$_2$$CH_2$(CH$_2$)$_5CH_3$, −CH$_2$$CH_2$(CH$_2$)$_7CH_3$), 22.4 (−($CH_2$)$_6$$CH_2CH_3$, −($CH_2$)$_8$$CH_2CH_3$), 16.3 (−B(OCH$_2$)$_3CCH_3$), 13.2 (−(CH$_2$)$_7$$CH_3$, −(CH$_2$)$_9$$CH_3$).

HRMS (ESI): m/z calcd for $C_{37}H_{56}BO_3NBr^-$: 652.35366; found: 652.35591 [M–K]$^-$

Potassium 3-(6-bromo-9-(2-octyldecyl)-9*H*-carbazole-2-yl)triolborate

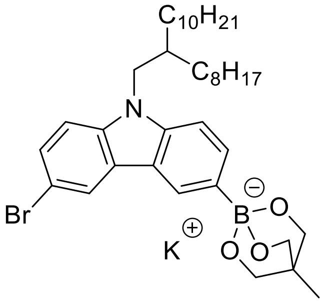

4.18 g, 97.2% yield: Brown solid

^{1}H NMR (400 MHz, DMSO-d_6): δ (ppm) 8.19 (d, J = 1.4 Hz, 1H, Ar–H), 8.12 (s, 1H, Ar–H), 7.58 (d, J = 8.2 Hz, 1H, Ar–H), 7.44-7.38 (m, 2H, Ar–H), 7.22 (d, J = 8.2 Hz, 1H, Ar–H), 4.14 (d, 2H, J = 7.3 Hz, −NCH_2CH−), 3.37 (s, 6H, −BOCH_2−), 2.00 (m, 1H, −NCH$_2$CH−), 1.15-1.24 (m, 32H, −CH$_2$(CH_2)$_7$CH$_3$, −CH$_2$(CH_2)$_9$CH$_3$), 0.84 (m, 6H, −(CH$_2$)$_7$CH_3, −(CH$_2$)$_9$CH_3), 0.59 (s, 3H, −BOCH$_2$CCH_3).

^{13}C NMR (100 MHz, DMSO-d_6): δ (ppm) 140.7 (Ar), 139.3 (Ar), 132.3 (Ar), 127.0 (Ar), 125.4 (Ar), 124.8 (Ar), 122.5 (Ar), 120.4 (Ar), 111.2, 110.6 (Ar), 107.6 (Ar), 72.7 ($-B(OCH_2)_3-$), 47.3 ($-NCH_2CH-$), 37.4 ($-NCH_2CH-$), 35.5 ($-B(OCH_2)_3CCH_3$), 31.5 ($-CH_2CH_2(CH_2)_5CH_3$, $-CH_2CH_2(CH_2)_7CH_3$), 29.5 ($-CH_2CH_2(CH_2)_4CH_2CH_3$, $-CH_2CH_2(CH_2)_6CH_2CH_3$), 26.3 ($-CH_2CH_2(CH_2)_5CH_3$, $-CH_2CH_2(CH_2)_7CH_3$), 22.7 ($-(CH_2)_6CH_2CH_3$, $-(CH_2)_8CH_2CH_3$), 17.0 ($-B(OCH_2)_3CCH_3$), 14.5 ($-(CH_2)_7CH_3$, $-(CH_2)_9CH_3$).

HRMS (ESI): *m/z* calcd for $C_{37}H_{56}BO_3NBr^-$: 652.35366; found: 652.35580 $[M–K]^-$

Synthesis of Bromo-9-(2-octyldecyl)-9*H*-carbazole-2-yl 4,4,5,5-tetramethyl-1,2,3dioxaborolane

Scheme S2. Synthesis of pinacol boronate monomers

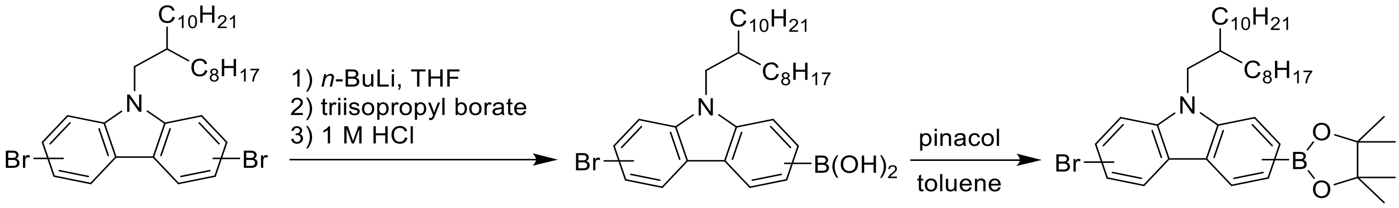

A general protocol is as follows: To a 500 mL three-necked round bottom flask, dibromo-9-alkyl-9*H*-carbazole (2,7-dibromo-9-(2-octyldecyl)-9*H*-carbazole or 3,6-dibromo-9-(2-octyldecyl)-9*H*-carbazole; 7.5 g, 12.4 mmol) was added and dried under vacuum at room temperature for 1.5 h. Dry-THF (100 mL) was introduced to this flask and cooled to −78 °C. *n*-BuLi (8.8 mL, 13.6 mmol, 1.6 mol L^{-1} in *n*-hexane) was injected slowly via a syringe, and the whole mixture was stirred for 3 h under argon atmosphere. Triisopropyl borate (4.3 mL, 18.6 mmol) was then added to this reaction mixture. The mixture was gradually brought back to room temperature and stirred for 12 h under argon atmosphere. The reaction was quenched by the addition of 1 mol L^{-1} HCl (30 mL). The solvent was removed by evaporation, and the residue was dissolved in CH_2Cl_2 and washed with brine. The organic layer was dried over Na_2SO_4 and evaporated completely.

To 500 mL flask containing toluene (280 mL), the obtained residue and pinacol (1.5 g, 13.0 mmol) were added and refluxed for 12 h. The solvent was removed by evaporation, and the residue was dissolved in diethylether. After wash with brine, the organic layer was dried over Na_2SO_4, and concentrated. The residue was purified by silica gel column chromatography (*n*-hexane/acetone/Et_3N = 94/4/2 (v/v/v)) to give bromo-9-alkyl-9*H*-carbazole-2-yl 4,4,5,5-tetramethyl-1,2,3dioxaborolane.

2-(7-Bromo-9-(2-octyldecyl)-9*H*-carbazole-2-yl)4,4,5,5-tetramethyl-1,2,3dioxaborolane

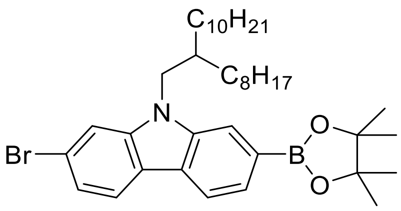

4.20 g, 56.8% yield: Yellow liquid

^{1}H NMR (400 MHz, $CDCl_3$): δ (ppm) 8.06 (d, *J* = 7.8 Hz, 2H, Ar–*H*), 7.87 (s, 1H, Ar–*H*), 7.69 (s, 1H, Ar–*H*), 7.56 (d, *J* = 7.8 Hz, 1H, Ar–*H*), 7.47 (d, *J* = 7.3 Hz, 1H, Ar–*H*), 4.14 (dd, 2H, *J* = 7.3, 2.3 Hz, $-NCH_2CH-$), 2.16 (m, 1H, $-NCH_2CH-$), 1.19–1.40 (m, 44H, $-CH(CH_2)_7CH_3$, $-CH(CH_2)_9CH_3$, $-BOC(CH_3)_2-$), 0.85–0.89 (m, 6H, $-(CH_2)_7CH_3$, $-(CH_2)_9CH_3$).

^{13}C NMR (100 MHz, $CDCl_3$): δ (ppm) 141.1 (Ar), 140.8 (Ar), 130.4 (Ar), 129.9 (Ar), 129.8 (Ar), 124.3 (Ar), 123.7 (Ar), 123.4 (Ar), 119.8 (Ar), 119.6 (Ar), 114.7 (Ar), 114.3 (Ar), 73.7 ($-BOC(CH_3)_2-$), 46.9 ($-NCH_2CH-$), 37.9 ($-NCH_2CH-$), 31.9 ($-CH_2(CH_2)_6CH_3$, $-CH_2$ $(CH_2)_8CH_3$), 29.7 ($-CH_2CH_2(CH_2)_4$ CH_2CH_3, $-CH_2CH_2(CH_2)_6CH_2CH_3$), 26.6 ($-CH_2CH_2(CH_2)_5CH_3$, $-CH_2CH_2(CH_2)_7CH_3$), 25.0 ($-BOC(CH_3)_2-$), 22.7 ($-(CH_2)_6CH_2CH_3$, $-(CH_2)_8CH_2CH_3$), 14.2 ($-(CH_2)_7CH_3$, $-(CH_2)_9CH_3$.

HRMS (ESI): *m*/*z* calcd for $C_{38}H_{59}BO_2NBrNa^+$: 674.37144; found: 674.37190 $[M+Na]^+$

3-(6-Bromo-9-(2-octyldecyl)-9*H*-carbazole-2-yl)4,4,5,5-tetramethyl-1,2,3dioxaborolane

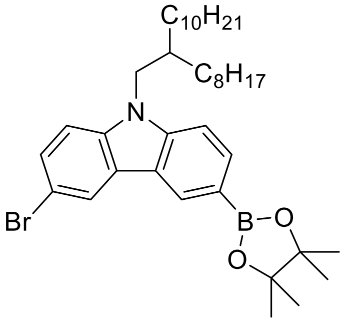

3.70 g, 46.8% yield: Yellow liquid

^{1}H NMR (400 MHz, $CDCl_3$): δ (ppm) 8.54 (s, 1H, Ar–*H*), 8.23 (d, *J* = 1.8 Hz, 1H, Ar–*H*), 7.91 (dd, *J* = 8.2, 0.9 Hz, 1H, Ar–*H*), 7.52-7.50 (m, 1H, Ar–*H*), 7.35 (d, *J* = 8.2 Hz, 1H, Ar–*H*), 7.25-7.22 (m, 1H, Ar–*H*), 4.12 (dd, 2H, *J* = 7.3, 2.3 Hz, −NCH_2CH−), 2.06 (m, 1H, −NCH_2C*H*−), 1.19–1.40 (m, 44H, −CH(CH_2)$_7$$CH_3$, −CH(C$H_2$)$_9$$CH_3$, −BOC(C$H_3$)$_2$−), 0.85–0.89 (m, 6H, −($CH_2$)$_7CH_3$, −($CH_2$)$_9CH_3$).
^{13}C NMR (100 MHz, $CDCl_3$): δ (ppm) 143.3 (Ar), 139.7 (Ar), 132.7 (Ar), 128.3 (Ar), 128.0 (Ar), 124.9 (Ar), 123.3 (Ar), 121.6 (Ar), 120.6 (Ar), 112.1 (Ar), 110.5 (Ar), 108.7 (Ar), 83.7 (−BO*C*(CH_3)$_2$−), 47.9 (−N*C*H_2CH−), 37.9 (−NCH_2*C*H−), 31.9 (−*C*H_2(CH_2)$_6$$CH_3$, −*C*$H_2$ (CH_2)$_8$$CH_3$), 29.7 (−$CH_2$$CH_2$(*C*$H_2$)$_4$ $CH_2$$CH_3$, −$CH_2$$CH_2$(*C*$H_2$)$_6$$CH_2$$CH_3$), 26.6 (−$CH_2$*C*$H_2$($CH_2$)$_5$$CH_3$, −$CH_2$*C*$H_2$($CH_2$)$_7$$CH_3$), 25.0 (−BOC(*C*$H_3$)$_2$−), 22.7 (−(*C*$H_2$)$_6$*C*$H_2$$CH_3$, −(*C*$H_2$)$_8$*C*$H_2$$CH_3$), 14.2 (−($CH_2$)$_7$*C*$H_3$, −($CH_2$)$_9$*C*$H_3$.

HRMS (ESI): *m*/*z* calcd for $C_{38}H_{59}BO_2NBrNa^+$: 674.37144; found: 674.37240 $[M+Na]^+$

Synthesis of iodobenzene-terminated polymethyl methacrylate

Scheme S3. Synthesis of iodobenzene-terminated poly(methyl methacrylate)

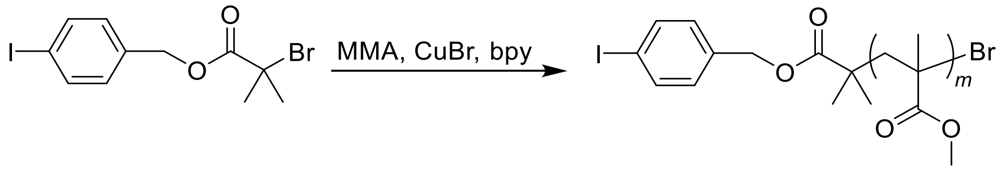

CuBr (15.0 mg, 105 μmol, 1.0 eq.) was evacuated for 30 min in a Schlenk flask and backfilled with argon. Methyl methacrylate monomer was passed through a basic Al_2O_3 column in order to remove the inhibitor. A mixture of methyl methacrylate monomer (1.05 g, 10.5 mmol, 100 eq.), 2,2'-bipyridyl (bpy; 16.3 mg, 105 μmol, 1.0 eq.), and 4-iodobenzyl 2-bromo-2-methylpropanoate (39.9 mg, 105 μmol, 1.0 eq.) were subjected to three freeze-pump-thaw cycles. Next, the liquid mixture was transferred to the Schlenk flask containing CuBr for the polymerization. The polymerization was performed in a preheated oil bath at 90 °C, and the monomer conversion was monitored by ^{1}H NMR at different polymerization time interval. The polymerization was terminated by bubbling air into the solution. The crude product was passed through a neutral Al_2O_3 column and eluted with THF to remove the catalyst. The mixture was purified by reprecipitation using THF as a good solvent and cold MeOH as a poor solvent to give iodobenzene-terminated polymethyl methacrylate (**PMMA−I**; 349 mg, 17.2% yield) as a white powder.

$M_{n,SEC}$ = 8,000 g mol^{-1} (THF); $M_{n,NMR}$ = 8,900 g mol^{-1} ($CDCl_3$), $Đ_M$ = 1.09.
^{1}H NMR ($CDCl_3$, 400 MHz): δ (ppm) 7.71-7.10 (m, Ar–*H*), 6.49-6.39 (m, Ar–*H*), 5.01 (s, –CH_2O–), 3.60 (s, O–CH_3), 2.07-1.81 (m, –CH_2–), 1.44-0.83 (m, –C(=O)C(CH_3)$_2$–, –CH_3).

Polymerization of potassium 3-(6-bromo-9-(2-octyldecyl)-9*H*-carbazole-2-yl)triolborate

Scheme S4. Polymerization of triolborate-type 3,6-carbazole monomer

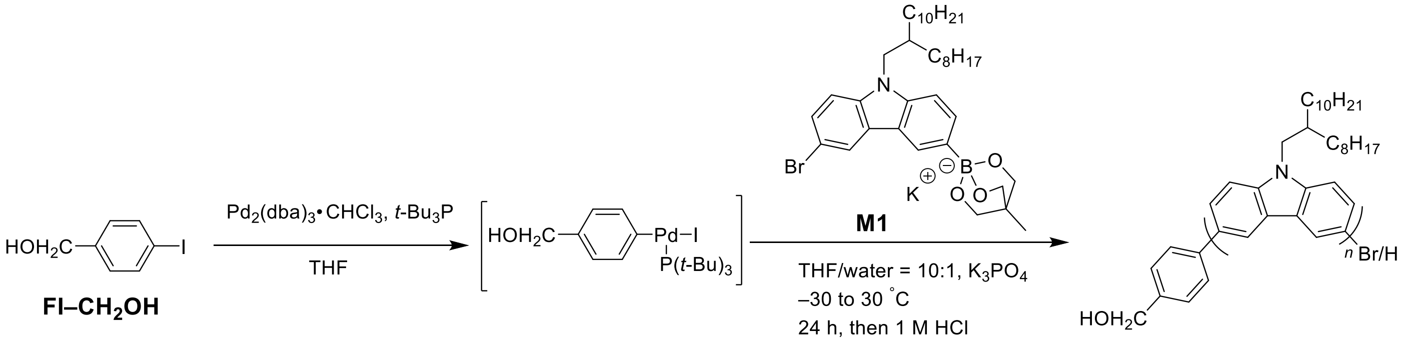

In the glovebox, 4-iodobenzyl alcohol (**FI−CH$_2$OH**; 30 μL, 1.4 μmol, as 0.050 mol L^{-1} stock solution in THF), $Pd_2(dba)_3$•$CHCl_3$ (0.40 mg, 0.43 μmol), and *t*-Bu_3P (6.0 μL, 3.2 μmol, as 0.5 mol L^{-1} stock solution in THF) were placed in a vial and dissolved in THF (200 μL), after stirring for 1 h at room temperature. The vial containing the stock solution of the Pd-initiator was sealed and taken out from the glovebox. In a 30 mL recovery flask, a mixture of THF (3.9 mL), deionized water (388 μL), potassium 3-(6-bromo-9-(2-octyldecyl)-9*H*-carbazole-2-yl)triolborate (**M1**; 30 mg, 0.043 mmol), and K_3PO_4 (0.46 mg, 2.2 μmol) were deoxygenated by argon bubbling at least for 1 h. The stock solution of the Pd-initiator was quickly added to the mixture under an argon atmosphere, and the entire mixture was vigorously stirred for 24 h at −10 °C. To the reaction mixture, 1 mol L^{-1} HCl (5 mL) was added to terminate the polymerization. The solvent was removed by evaporation, and the residue was dissolved in CH_2Cl_2 and washed with brine. The organic layer was dried over Na_2SO_4 and concentrated. The mixture was filtered, and the filtrate was evaporated under reduced pressure. The resulting solution was concentrated and precipitated using THF as a good solvent and cold poor solvent (MeOH/acetone = 2/1 (v/v)) to give **HOCH$_2$−3,6-PCz**. The polymerization results are listed in **Table 1**.

11.6 mg, 60.1% yield: Yellow solid.
$M_{n,SEC}$ = 6,300 g mol^{-1} (THF); $M_{n,NMR}$ = 6,700 g mol^{-1} ($CDCl_3$), $Đ_M$ = 1.19.
$T_{d,\,10\%}$ = 330 °C, Abs. (λ_{max}) = 319 nm ($CHCl_3$); Emi. (λ_{max}) = 509 nm ($CHCl_3$).
^{1}H NMR (400 MHz, $CDCl_3$): δ (ppm) 8.62–8.23 (m, Ar–*H*), 7.93–7.77 (m, Ar–*H*), 7.54–7.36 (m, Ar–*H*), 4.75 (s, –C*H*$_2$O–), 4.14 (s, −NC*H*$_2$CH−), 2.33–1.97 (m, −NCH$_2$C*H*−), 1.49–1.06 (br, −CH$_2$(C*H*$_2$)$_7$CH$_3$, −CH$_2$(C*H*$_2$)$_9$CH$_3$), 0.91–0.76 (m, −(CH$_2$)$_7$C*H*$_3$, −(CH$_2$)$_9$C*H*$_3$).

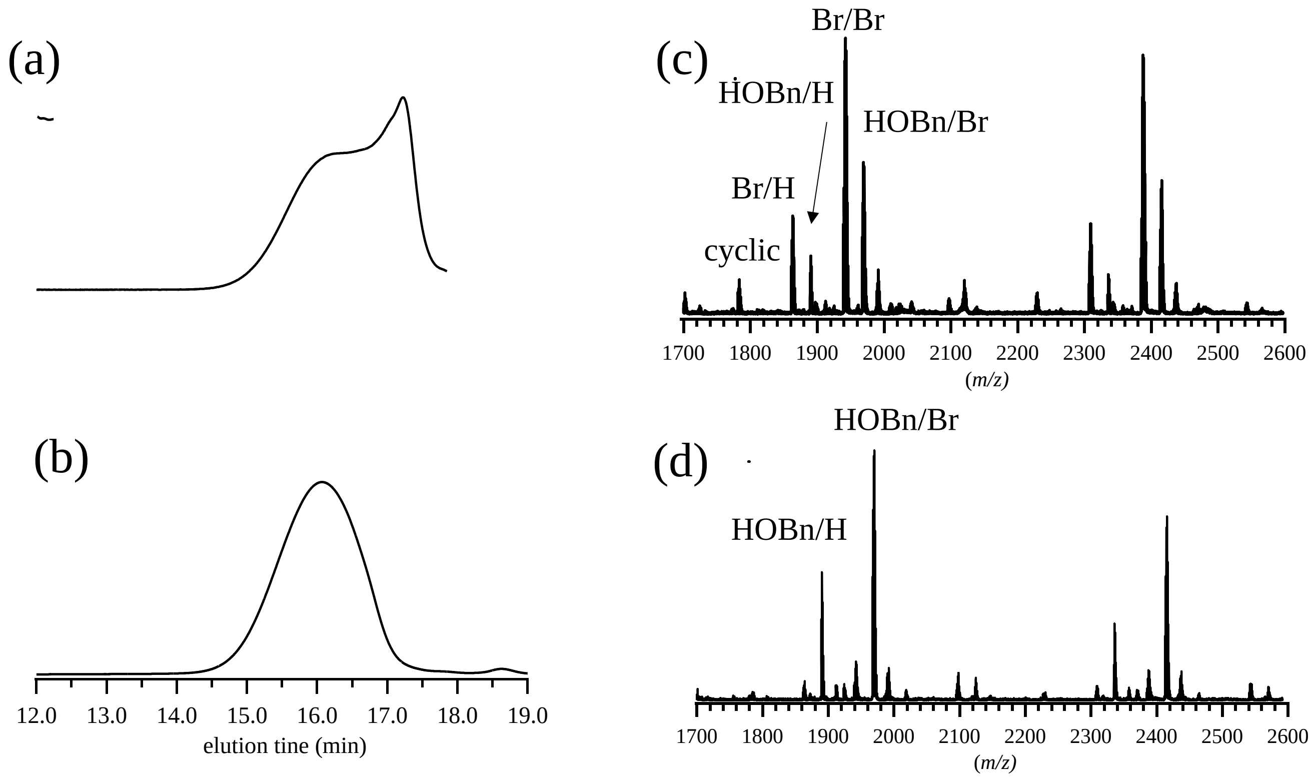

Figure S1. (a) SEC trace of 3,6-PCz (run 1, Table 1) detected by RI detector (eluent, THF; flow rate, 1.0 mL min^{-1}). (c) MALDI-TOF mass spectrum of 3,6-PCz obtained from run 1 (Table 1). (b and d) SEC trace and MALDI-TOF mass spectrum of 3,6-PCz obtained from run 1 after preparative SEC.

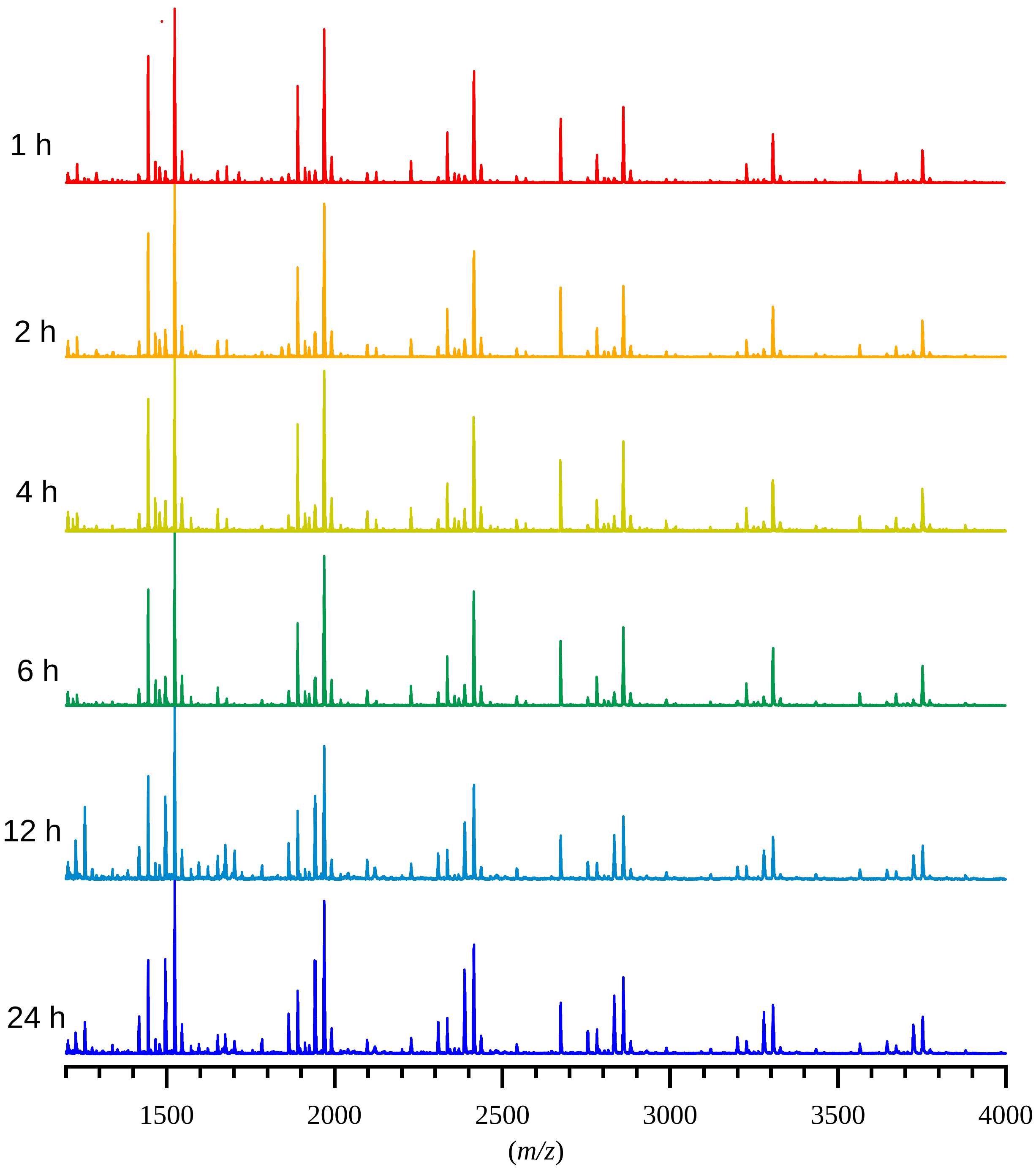

Figure S2. Expanded MALDI-TOF mass spectra ranging from 1200 to 4000 Da of 3,6-PCz.

Polymerization of potassium 2-(7-bromo-9-(2-octyldecyl)-9*H*-carbazole-2-yl)triolborate

Scheme S5. Polymerization of triolborate-type 2,7-carbazole monomer

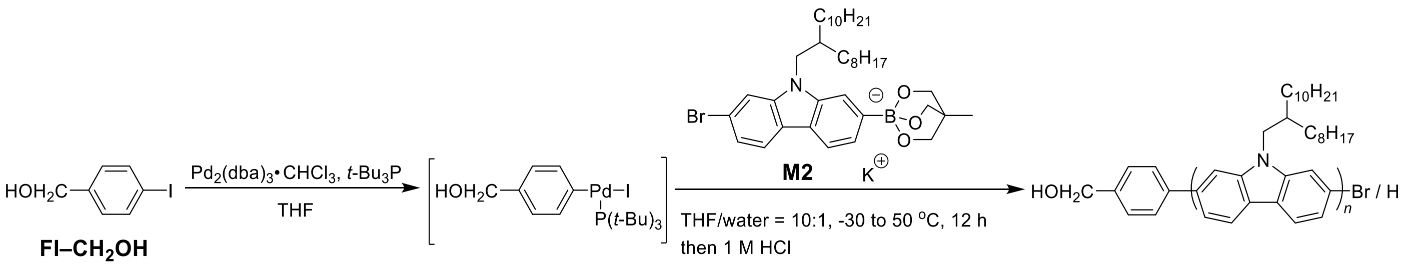

In the glovebox, 4-iodobenzyl alcohol (**FI−CH_2OH**; 30 μL, 1.4 μmol, as 0.050 mol L^{-1} stock solution in THF), $Pd_2(dba)_3$•$CHCl_3$ (0.40 mg, 0.43 μmol), and *t*-Bu_3P (6.0 μL, 3.2 μmol, as 0.5 mol L^{-1} stock solution in THF) were placed in a vial and dissolved in THF (200 μL), after stirring for 1 h at room temperature. The vial containing the stock solution of the Pd-initiator was sealed and taken out from the glovebox. In a 30 mL recovery flask, a mixture of THF (3.9 mL), deionized water (388 μL), potassium 2-(7-bromo-9-(2-octyldecyl)-9*H*-carbazole-2-yl)triolborate (**M2**; 30 mg, 0.043 mmol), and K_3PO_4 (0.46 mg, 2.2 μmol) were deoxygenated by argon bubbling at least for 1 h. The stock solution of the Pd-initiator was quickly added to the mixture under an argon atmosphere, and the entire mixture was vigorously stirred for 12 h at −10 °C. To the reaction mixture, 1 mol L^{-1} HCl (5 mL) was added to terminate the polymerization. The solvent was removed by evaporation, and the residue was dissolved in CH_2Cl_2 and washed with brine. The organic layer was dried over Na_2SO_4 and concentrated. The mixture was filtered, and the filtrate was evaporated under reduced pressure. The resulting solution was concentrated and precipitated using THF as a good solvent and cold poor solvent (MeOH/acetone = 2/1 (v/v)) to give **HOCH_2−2,7-PCz**. The polymerization results are listed in **Table 1**.

14.4 mg, 74.8% yield: Yellow solid.
$M_{n,SEC}$ = 3,700 g mol^{-1} (THF); $M_{n,NMR}$ = 5,080 g mol^{-1} ($CDCl_3$), $Đ_M$ = 1.23.
$T_{d,\,10\%}$ = 428 °C; T_g = 122 °C, Abs. (λ_{max}) = 379, 274 nm ($CHCl_3$); Emi. (λ_{max}) = 419 nm ($CHCl_3$).
^{1}H NMR (400 MHz, $CDCl_3$): δ (ppm) 8.16–7.84 (m, Ar–*H*), 7.77–7.50 (m, Ar–*H*), 4.75 (s, –CH_2O–), 4.14 (s, −NCH_2CH−), 2.33–1.97 (m, −NCH_2C*H*−), 1.49–1.06 (br, −CH_2(CH_2)$_7$$CH_3$, −$CH_2$(C$H_2$)$_9$$CH_3$), 0.91–0.76 (m, −($CH_2$)$_7CH_3$, −($CH_2$)$_9CH_3$).

Evaluation of the living nature of the SCTP of triolborate salt monomer

Scheme S6. Kinetic study for SCTP of M2

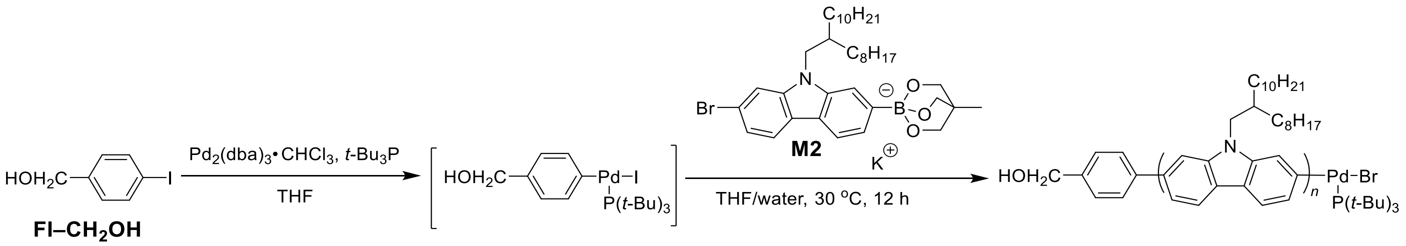

In the glovebox, **FI−CH₂OH** (192 μL, 9.62 μmol, as 0.5 mol L^{-1} stock solution in THF), $Pd_2(dba)_3$•$CHCl_3$ (3.0 mg, 2.87 μmol), and *t*-Bu_3P (42.3 μL, 21.1 μmol, as 0.5 mol L^{-1} stock solution in THF) were placed in a vial and dissolved in THF (3 mL), then stirring for 1 h at room temperature. The vial containing the stock solution of the Pd-initiator was sealed and taken out from the glovebox. In a 100 mL recovery flask, a mixture of THF (23 mL), deionized water (2.6 mL), **M2** (200 mg, 0.289 mmol), and K_3PO_4 (3.06 mg, 14.4 μmol) were deoxygenated by argon bubbling at least for 1 h. The stock solution of the Pd-initiator was quickly added to the mixture under an argon atmosphere, and the entire mixture was vigorously stirred for 12 h at 30 °C. A small aliquot (2 mL) of the reaction mixture was collected at 1.0, 3.0, 6.0, and 12 h. Each aliquot was quenched with 1 mol L^{-1} HCl solution and extracted with CH_2Cl_2. The separated organic layer was evaporated under reduced pressure to get a residue. Half of the residue was dissolved in $CDCl_3$ to determine the conversion of monomer by ^{1}H NMR (conversions of 42.3%, 63.3%, 85.6%, and 98.5% were observed for 1.0, 3.0, 6.0, and 12 h, respectively). The other half of the residue was dissolved in THF, and the solution was filtered. The filtrate was analyzed by SEC to determine the *M*n and $Đ_M$ values of the polymers. The $M_{n,SEC}$ ($Đ_M$) values of each polymer initiated by $Pd_2(dba)_3$•$CHCl_3$/*t*-Bu_3P/**FI−CH₂OH**/K_3PO_4 for 1.0, 3.0, 6.0, and 12 h were 3100 (1.20), 5300 (1.23), 7500 (1.30), and 9200 g mol^{-1} (1.31) respectively. The polymerization results are listed in **Table S1**.

Table S1. Monomer conversion, molecular weight, and dispersity at each reaction time[a]

reaction time (h)	conversion (%)	$M_{n,SEC}$[b] (g mol^{-1})	$Đ_M$[b]	$M_{n,NMR}$[c] (g mol^{-1})
1.0	42.3	3,100	1.20	4,300
3.0	63.3	5,300	1.23	7,300
6.0	85.6	7,500	1.30	10,000
12	98.5	9,200	1.31	11,500

[a]Polymerization conditions: Ar atmosphere; solvent, THF/water (v/v) = 10:1; [**M2**]$_0$ = 10 mmol L^{-1}; [**M2**]$_0$/[**FI−CH₂OH**]$_0$/[$Pd_2(dba)_3$•$CHCl_3$]/[*t*-Bu_3P]/[K_3PO_4] = 30:1:0.3:2.2:1.5. [b]Determined by SEC (PSt standards, THF, 40 °C). [c]Determined by ^{1}H NMR spectrum in $CDCl_3$.

Post-polymerization experiment for the SCTP of triolborate

Scheme S7. Block copolymerization experiment to confirm living nature of SCTP of M2

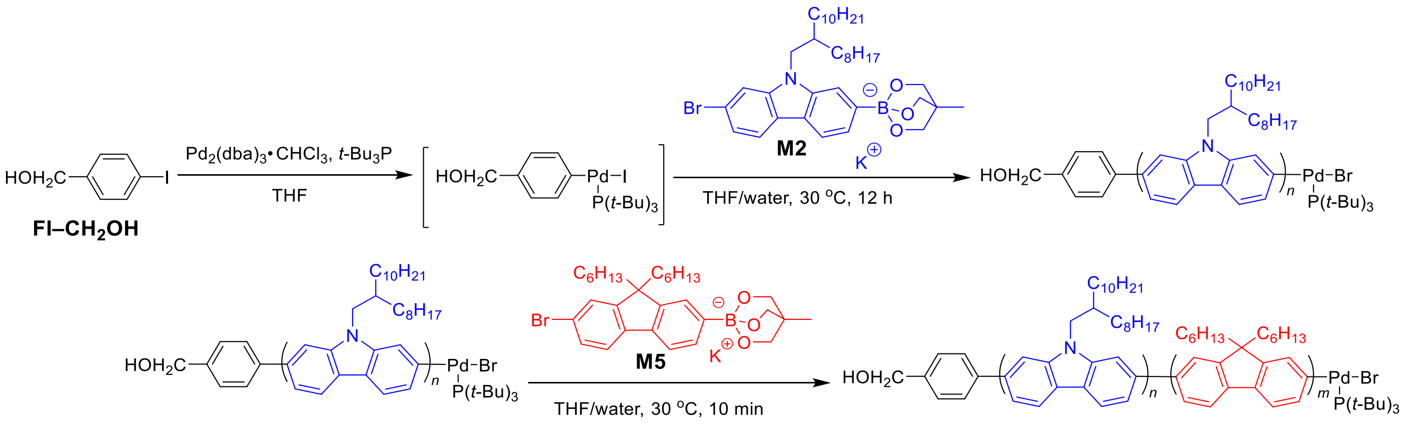

In the glovebox, **FI−CH₂OH** (1.15 mL, 0.58 mmol, as 0.5 mol L^{-1} stock solution in THF), $Pd_2(dba)_3$•$CHCl_3$ (178.6 mg, 0.173mmol), and *t*-Bu_3P (2.53 mL, 1.27 mmol, as 0.5 mol L^{-1} stock solution in THF) were placed in a vial and dissolved in THF (15 mL), then stirring for 1 h at room temperature. The vial containing the stock solution of the Pd-initiator was sealed and taken out from the glovebox. In a 100 mL recovery flask, a mixture of THF (3.9 mL), deionized water (388 μL), **M2** (30 mg, 0.043 mmol), and K_3PO_4 (0.46 mg, 2.2 μmol) were deoxygenated by argon bubbling at least for 1 h. The stock solution of the Pd-initiator was quickly added to the mixture under an argon atmosphere, and the entire mixture was vigorously stirred for 12 h at 30 °C. After withdrawing a small aliquot of the mixture, **M5** (25 mg, 0.043 mmol) was then added, and the whole mixture was stirred for 10 min at 30 °C. The final products was obtained as a yellow solid.

2,7-PCz-*b*-PF

30 mg, 88.6% yield: Yellow solid.

$M_{n,SEC}$ = 7,800 g mol^{-1} (THF); $M_{n,NMR}$ = 9,400 g mol^{-1} ($CDCl_3$), $Đ_M$ = 1.38.

^{1}H NMR (400 MHz, $CDCl_3$): δ (ppm) 8.16–7.50 (m, Ar–*H*), 4.75 (s, –CH_2O–), 4.14 (s, −NCH_2CH−), 2.33–1.97 (m, −NCH$_2$C*H*−, Ar–(CH_2(CH$_2$)$_4$CH$_3$)$_2$), 1.49–1.06 (br, −CH_2−), 0.91–0.66 (m, −CH_3).

Molecular weight control

Scheme S8. Molecular weight control of 2,7-PCz

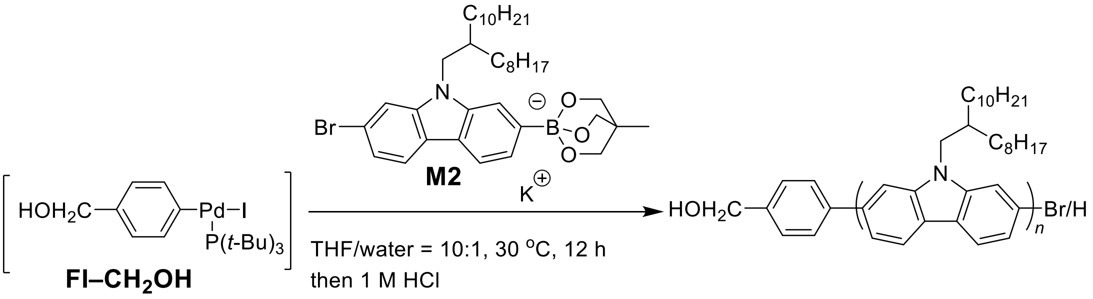

The SCTP of **M2** was conducted with the optimized condition while varying the [**M2**]$_0$/[**FI−CH$_2$OH**]$_0$ ratio (30:1, 60:1, and 90:1) for aiming at synthesizing the higher molecular weight PFs. The final product of **HOCH$_2$−PF** was obtained as a yellow solid. The polymerization results are listed in **Table 1**.

Polymerization of bromo-9-(2-octyldecyl)-9*H*-carbazole-2-yl 4,4,5,5-tetramethyl-1,2,3dioxaborolane

Scheme S9. Polymerization of pinacolboronate-type carbazole monomers

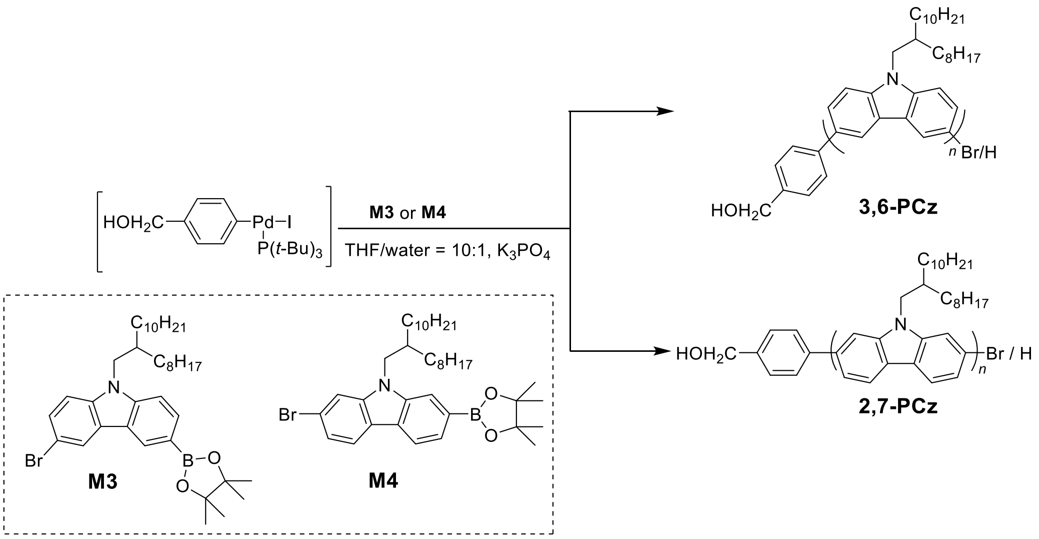

The PCzs were synthesized by the SCTP of pinacolboronate-type carbazole monomer (**M3** or **M4**; 3.45 mL, 0.86 mmol) with **HOCH$_2$–PF** (115 μL, 57.5 μmol, as 0.50 mol L^{-1} stock solution in THF), $Pd_2(dba)_3$•$CHCl_3$ (17.9 mg, 17.2 μmol), *t*-Bu_3P (253 μL, 127 μmol, as 0.5 mol L^{-1} stock solution in THF) in a mixture of THF (86 mL), K_3PO_4 (18.3 mg, 86.3 μmol), and deionized water (8.6 μL).

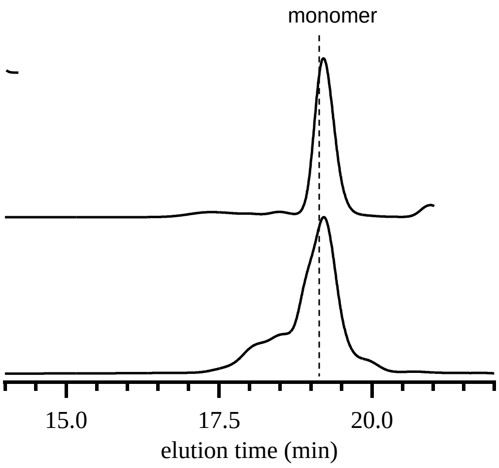

Figure S3. SEC traces of crude reaction mixture for the SCTP of **M3** (upper) and **M4** (lower) (eluent, THF; flow rate, 1.0 mL min^{-1}).

Synthesis of PCz or PF containing random copolymers

Scheme S10. Random copolymerization of M1/M2, M1/M5, and M2/M5

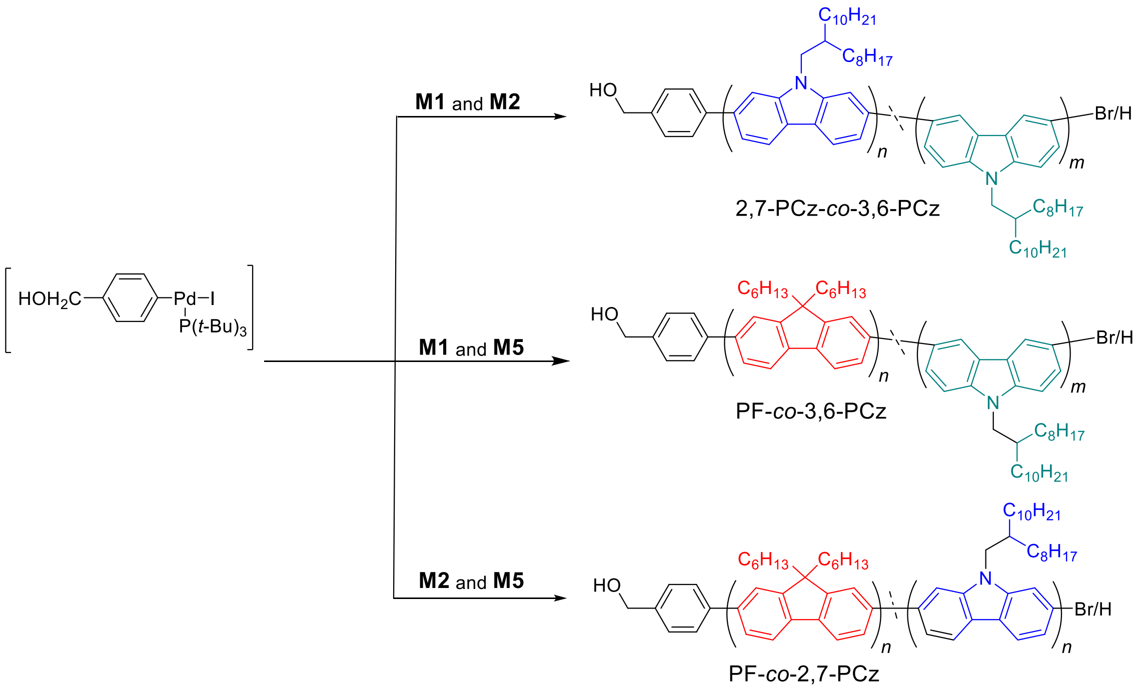

The PCz- or PF-containing random copolymers were synthesized by the SCTP of triolborate-type monomer (21.7 μmol) and the other monomer (21.7 μmol) with **FI−CH$_2$OH** (58 μL, 28.9 μmol, as 0.50 mol L^{-1} stock solution in THF), $Pd_2(dba)_3$•$CHCl_3$ (0.90 mg, 8.7 μmol), *t*-Bu$_3$P (127 μL, 63.5 μmol, as 0.50 mol L^{-1} stock solution in THF) in a mixture of THF (3.88 mL), K_3PO_4 (0.92 mg, 4.3 μmol), and deionized water (388 μL). The final products were obtained as a yellow solid. The polymerization results are listed in **Table 2**.

2,7-PCz-*co*-3,6-PCz

28 mg, 72.6% yield: Yellow solid.

$M_{n,SEC}$ = 7,800 g mol^{-1} (THF); $M_{n,NMR}$ = 11,500 g mol^{-1} ($CDCl_3$), $Đ_M$ = 1.49.

$T_{d,\,10\%}$ = 337 °C, Abs. (λ_{max}) = 354, 312 nm ($CHCl_3$); Emi. (λ_{max}) = 413 nm ($CHCl_3$).

^{1}H NMR (400 MHz, $CDCl_3$): δ (ppm) 8.62–8.23 (m, Ar–*H*), 8.16–7.84 (m, Ar–*H*), 7.77–7.36 (m, Ar–*H*), 4.75 (s, –C*H*$_2$O–), 4.14 (s, −NC*H*$_2$CH−), 2.33–1.97 (m, −NCH$_2$C*H*−), 1.49–1.06 (br, −CH$_2$(C*H*$_2$)$_7$CH$_3$, −CH$_2$(C*H*$_2$)$_9$CH$_3$), 0.91–0.76 (m, −(CH$_2$)$_7$C*H*$_3$, −(CH$_2$)$_9$C*H*$_3$).

PF-*co*-3,6-PCz

25 mg, 74.6% yield: Yellow solid.

$M_{n,SEC}$ = 9,300 g mol^{-1} (THF); $M_{n,NMR}$ = 12,300 g mol^{-1} ($CDCl_3$), $Đ_M$ = 1.38.

$T_{d,\,10\%}$ = 362 °C, Abs. (λ_{max}) = 362, 318 nm ($CHCl_3$); Emi. (λ_{max}) = 417 nm ($CHCl_3$).

^{1}H NMR (400 MHz, $CDCl_3$): δ (ppm) 8.62–8.23 (m, Ar–*H*), 8.16–7.84 (m, Ar–*H*), 7.77–7.36 (m, Ar–*H*), 4.75 (s, –C*H*$_2$O–), 4.14 (s, −NC*H*$_2$CH−), 2.33–1.97 (m, −NCH$_2$C*H*−, Ar–(C*H*$_2$(CH$_2$)$_4$CH$_3$)$_2$), 1.49–1.06 (br, −C*H*$_2$−), 0.91–0.66 (m, −C*H*$_3$).

PF-*co*-2,7-PCz

26 mg, 76.1% yield: Yellow solid.

$M_{n,SEC}$ = 7,800 g mol^{-1} (THF); $M_{n,NMR}$ = 12,500 g mol^{-1} ($CDCl_3$), $Đ_M$ = 1.48.

$T_{d,\,10\%}$ = 347 °C, T_g = 167 °C, Abs. (λ_{max}) = 378, 272 nm ($CHCl_3$); Emi. (λ_{max}) = 419 nm ($CHCl_3$).

^{1}H NMR (400 MHz, $CDCl_3$): δ (ppm) 8.16–7.50 (m, Ar–*H*), 4.75 (s, –CH_2O–), 4.14 (s, −NCH_2CH−), 2.33–1.97 (m, −NCH_2CH−, Ar–($CH_2(CH_2)_4CH_3)_2$), 1.49–1.06 (br, −CH_2−), 0.91–0.66 (m, −CH_3).

Synthesis of 2,7-PCz-containing diblock copolymers using macroinitiators

Scheme S11. Synthesis of PSt-*b*-2,7-PCz and PMMA-*b*-2,7-PCz by SCTP using macroinitiators

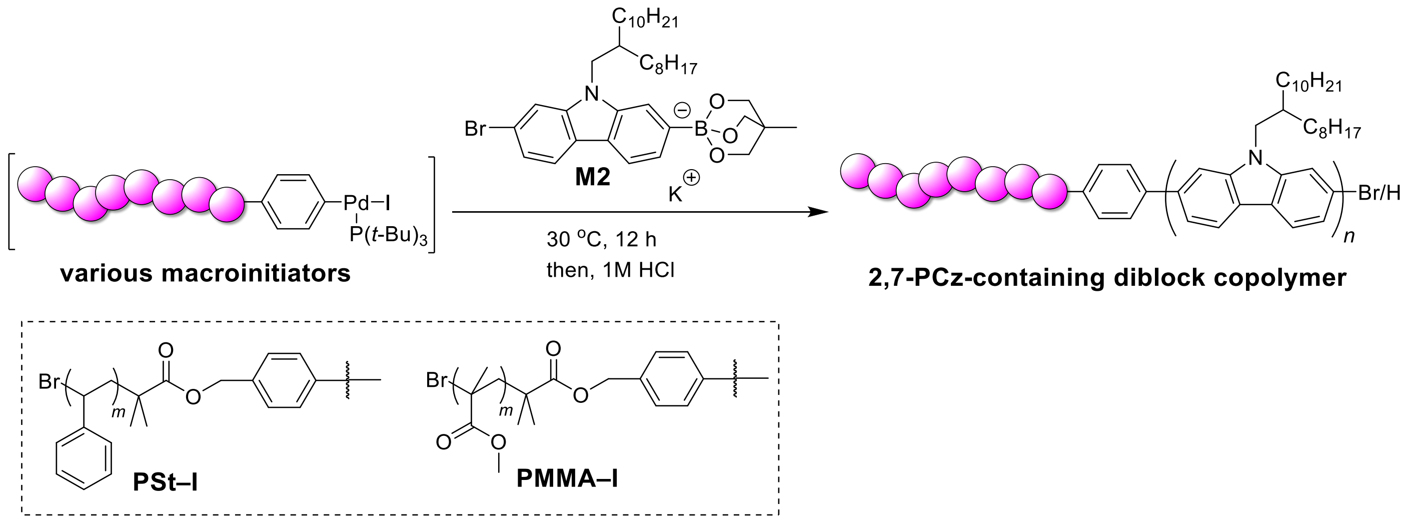

The 2,7-PCz-containing diblock copolymers were synthesized by the SCTP of **M2** (30 mg, 43.3 μmol) with the macroinitiators (2.89 μmol, 1.0 eq.; **PSt− I**; $M_{n,NMR}$; 8,300 g mol^{-1}, $Đ_M$; 1.24 and **PMMA−I**, $M_{n,\ NMR}$ = 8,900, $Đ_M$ = 1.09), $Pd_2(dba)_3$•$CHCl_3$ (3.6 mg, 3.47 μmol, 1.2 eq.), and *t*-Bu_3P (51 μL, 25.4 μmol, as 0.5 mol L^{-1} stock solution in THF, 8.8 eq.) in a mixture of THF (3.84 mL), K_3PO_4 (0.74 mg, 3.4 μmol, 1.2 eq.), and deionized water (0.80 μL). The resulting solution was concentrated and precipitated using THF as a good solvent and cold mixed solvent of MeOH/acetone (= 1/1 (v/v)) as a poor solvent to give the 2,7-PCz-containing diblock copolymers. The final products were obtained as a yellow solid.

PSt-*b*-2,7-PCz

30 mg, 78.6% yield: Yellow solid.

$M_{n,SEC}$ = 13,600 g mol^{-1} (THF); $M_{n,NMR}$ = 15,500 g mol^{-1} ($CDCl_3$), $Đ_M$ = 1.31.

^{1}H NMR (400 MHz, $CDCl_3$): δ (ppm) 8.16–7.84 (m, Ar–*H*), 7.77–7.50 (m, Ar–*H*), 6.49–6.39 (m, Ar–*H*), 5.01 (s, –CH_2O–), 4.14 (s, −NCH_2CH−), 2.33–1.97 (m, −NCH_2C*H*−, Ar−CH_2−), 1.92 (s, –CH_3), 1.49–1.06 (br, −CH_2(CH_2)$_7$$CH_3$, −$CH_2$(C$H_2$)$_9$$CH_3$), 0.91–0.76 (m, −($CH_2$)$_7CH_3$, −($CH_2$)$_9CH_3$).

PMMA-2,7-PCz

33 mg, 73.5% yield: Yellow solid.

$M_{n,SEC}$ = 12,900 g mol^{-1} (THF); $M_{n,NMR}$ = 15,700 g mol^{-1} ($CDCl_3$), $Đ_M$ = 1.33.

^{1}H NMR (400 MHz, $CDCl_3$): δ (ppm) 8.16–7.84 (m, Ar–*H*), 7.77–7.10 (m, Ar–*H*), 6.49-6.39 (m, Ar–*H*), 5.01 (s, –CH_2O–), 3.60 (s, O–CH_3), 4.14 (s, −NCH_2CH−), 2.33–1.97 (m, −NCH_2C*H*−, –CH_2–), 1.49–1.06 (br, −CH_2(CH_2)$_7$$CH_3$, −$CH_2$(C$H_2$)$_9$$CH_3$, –C(=O)C(C$H_3$)$_2$–), 0.91–0.76 (m, −C$H_3$).

Reference

1. A. Y. Fedorov, A. A. Shchepalov, A. V. Bol´shakov, A. S. Shavyrin, Y. A. Kurskii, J. P. Finet, and S. V. Zelentsov, *Russian chemical bulletin*, 2004, **53**, 370−375.
2. X. Zhu, S. R. Bheemireddy, S. V. Sambasivarao, P. W. Rose. R. T. Guzman. A. G. Waltner, K. H. DuBay, and K. N. Plunkett, *Macromolecules*, 2016, **49**, 127−133.
3. A. Drewniak, M. D. Tomcyk, L. hanusek, A. Mielanczyk, Krzytof Walczak, P. Nitschke, B. Hajduk, and P. Ledwon, *Polymers*, 2018, **10**, 487−501.
4. S. Kobayashi, K. Fujiwara, D.-H. Jiang, T. Yamamoto, K. Tajima, Y. Yamamoto, T. Isono, and T. Satoh, *Poly. Chem*., 2020, **11**, 6832−6839.
5. S. Zalesskiy and V. P. Ananikov, *Organometallics*, 2012, **31**, 2302−2309.
